# Effectiveness and Characteristics of Work Participation Interventions for Adults with Musculoskeletal Upper Limb Conditions: A Systematic Review

**DOI:** 10.1007/s10926-024-10251-6

**Published:** 2024-12-05

**Authors:** Lisa Newington, Daniel Ceh, Fiona Sandford, Vaughan Parsons, Ira Madan

**Affiliations:** 1 Barts Bone and Joint Health, Blizard Institute, Queen Mary University of London, First Floor Abernethy Building, 2 Newark Street, London, E1 2AT UK; 2 Hand Therapy, Barts Health NHS Trust, London, UK; 3 London Centre for Work and Health, London, UK; 4 Hand Therapy, Guy’s and St Thomas’ NHS Foundation Trust, London, UK; 5 School of Biomedical Engineering and Imaging Sciences, School of Life Couse and Population Sciences, King’s College London, London, UK; 6 Occupational Health, Safety and Wellbeing Service, Guy’s and St Thomas’ NHS Foundation Trust, London, UK; 7 Faculty of Life Sciences and Medicine, King’s College London, London, UK

**Keywords:** Work participation, Return to work, Fit note, Upper limb, Rehabilitation, Systematic review

## Abstract

**Purpose:**

To systematically identify and evaluate interventions to improve work participation for adults with upper limb musculoskeletal conditions, and explore contextual factors and mechanisms that suggest how the intervention is effective, for whom, and in what setting.

**Methods:**

The review protocol was pre-registered with PROSPERO (CRD42023433216). Eligible studies met the following criteria. *Population* adults (aged ≥ 18 years), with musculoskeletal upper limb conditions including traumatic and non-traumatic presentations. *Intervention* strategies aimed at enhancing work participation. *Outcomes* measures including return to work, increased work duties or hours, and work functioning. *Study design* randomised and non-randomised experimental studies, mixed methods, qualitative studies, and case series. Two reviewers independently screened, extracted data, and completed quality appraisal. Interventions were described using TIDieR and the data presented as a narrative synthesis.

**Results:**

Twenty-two studies were included. Interventions were categorised into three groups: multimodal or multidisciplinary (*n* = 13), ergonomic (*n* = 4), and exercise (*n* = 5). Eight interventions were primarily delivered in the workplace and 14 in healthcare settings. Four outcome domains were reported: return to work (*n* = 18), self-reported work function (*n* = 4), work productivity (*n* = 5), and work-related costs (*n* = 2). Only exercise interventions showed consistent statistically significant benefits. Heterogeneity in outcomes prevented formal meta-analysis. Only five studies were rated as high quality.

**Conclusions:**

There is insufficient evidence to recommend specific work participation interventions for adults with upper limb musculoskeletal systems. No studies explored the impact of Fit Notes or other formal work guidance documentation.

**Supplementary Information:**

The online version contains supplementary material available at 10.1007/s10926-024-10251-6.

## Introduction

Musculoskeletal disorders (MSDs) comprise a variety of diagnoses, including traumatic injuries, inflammatory conditions, and conditions of musculoskeletal pain, such as non-specific arm pain. MSDs are the highest contributor to the global need for rehabilitation [1] and are a common cause of work-related disability and sickness absence [2, 3]. There is broad consensus that supporting people with MSDs to remain in or return to work has positive impacts for their health and wellbeing [4], and has important economic benefits for individuals and society.

Rehabilitation clinicians, including physiotherapists and occupational therapists, are well placed to provide work participation support and guidance for their patients, but current best practice is yet to be established. Much of the existing research on MSDs and work has focussed on low back pain and suggests that workplace interventions [5], multidisciplinary biopsychosocial rehabilitation [6], and programmes of graded activity [7] are beneficial. The addition of a vocational advice service to primary care musculoskeletal consultations has been shown to significantly reduce work absence compared to usual practice [8].

A Cochrane review attempted to assess the impact of vocational rehabilitation for individuals with traumatic upper limb conditions, but found no eligible studies [9]. Existing reviews have explored the impact of workplace-based interventions [10], occupational therapy rehabilitation for work-related injuries [11], and early work participation interventions for adults with regional MSDs, including the upper limb [12, 13]. However, all found a lack of high-quality evidence and were unable to make clear treatment recommendations.

A 2022 UK legislative change enables physiotherapists and occupational therapists to issue Fit Notes for their patients [14]. This is formal documentation for recommended modifications to work duties, hours or schedule, or a period of sick leave, and was previously only certifiable by medical doctors. However, there has been low uptake [15, 16]. Only 25% of UK hand therapists reported issuing a fit note in 2023 [16], and surveys of hand therapists in the UK and Australia have found low confidence in issuing work recommendations for patients, and low satisfaction with the work participation support that is currently provided in practice [16, 17].

The current review was developed by clinical hand therapists to answer the question: what interventions enhance work participation for individuals with upper limb musculoskeletal conditions? Knowing that previous reviews had provided inconclusive evidence in related areas, a secondary purpose of this review was to explore the characteristics of the interventions and possible contextual factors and mechanisms that might determine how the intervention is effective, for whom, and in what setting.

## Methods

The review protocol was pre-registered with PROSPERO [18] and completed using the PRISMA and SWiM guidelines [19, 20] (Appendix 1). The database searches were undertaken on 27/07/23, and the grey literature searches on 23/01/2024. Searches were conducted in English language. Eligible studies were those reporting work-relevant outcomes for any type of work participation intervention for adults with upper limb musculoskeletal injuries or conditions. The upper limb was defined as hand to shoulder. Mixed populations including conditions affecting the hand to neck were included; isolated neck conditions were excluded. Diagnostic method was not pre-specified but was recorded as part of the review. Work participation was defined as return to work, increased work duties, hours or productivity, or reduced sickness absence. Eligibility criteria are summarised in Table 1.

**Table 1:** Review eligibility criteria

Inclusion	Exclusion
*Participant population* - Adults ≥ 18 years of age - Upper limb musculoskeletal injuries (i.e. to bone, tendon, ligament, peripheral nerves, or other soft tissue injuries) or acquired upper limb musculoskeletal conditions (e.g. osteoarthritis, inflammatory conditions, chronic pain) - In paid work, or attempting to commence or return to paid work through the study intervention	*Participant population* - Aged < 18 years - Injuries or conditions not affecting the upper limb - Issues of the central nervous system affecting the upper limb (e.g. stroke, Parkinson’s disease, traumatic brain injury) - Working in voluntary or unpaid roles
*Intervention* - Any strategy delivered with the aim of supporting return to work, reducing sickness absence or increasing work duties, hours or productivity	*Intervention* - Strategies without a specific work-related aim, for example, to improve pain, range of movement, or grip strength
*Outcomes* - Changes to work participation, e.g. return to work, reduced sickness absence, increased work hours, duties or productivity, work-related costs - Self-reported work functional ability	*Outcomes* - No work-related outcome reported
*Study design* - Quantitative studies including randomised controlled trials, quasi-experimental studies, cohort studies, and case series - Qualitative studies including interviews and focus groups - Mixed methods studies	*Study design* - Case studies - Systematic or other reviews - Editorials, opinion pieces, or letters - Conference abstracts

### Search Strategy

Search locations included six healthcare databases (Medline, PsychInfo, EMCARE, AMED, CINAHL, and PEDro) and four trials databases (ClinicalTrials.gov, ClinicalTrialsRegister.eu, Cochrane Central Register of Controlled Trials, and ISRCTN registry). Search format included title, abstract, and keywork searches with exploded medical subject headings, where available. The search strategy was piloted and refined in Medline (Appendix 2). Searches were completed from inception to July 2023.

Additionally, two Google searches were completed in a naïve browser for ‘work participation and upper limb’ and ‘upper extremity conditions and work’ (23/01/2024). The first 40 hits were screened for each phrase. References of identified systematic reviews and eligible articles were hand searched. All literature searching was completed by the lead author, with review by DC. Search findings were exported to Rayyan (Rayaan.ai) and duplicates removed before screening.

Title and abstract, and full text screening were independently performed by two reviewers (LN and DC). Cases of disagreement were discussed and resolved, with review and input by VP and FS.

### Data Extraction and Methodological Quality Assessment

Data were extracted using a pre-piloted electronic proforma that had been developed and reviewed by the whole team. Data extraction items are listed in Appendix 3. Methodological quality assessment was completed using the Johanna Briggs Institute (JBI) critical appraisal tool for randomised controlled trials, quasi-experimental studies, case series, and economic evaluations [21]. Additionally, the mixed methods critical appraisal tool [22] was used to appraise mixed methods studies. No purely qualitative studies were included and therefore a qualitative critical appraisal tool was not required. Quality assessment was completed independently by LN and DC and disagreements were resolved by discussion. A summary score was presented for each paper based on the lowest rating achieved for any one item. Scoring was not used to direct review eligibility.

### Data Synthesis and Reporting

The analysis was planned as a narrative synthesis because preliminary searching identified wide variation in reported work-related outcomes. Studies were grouped based on the nature of the intervention, which was categorised during data extraction and agreed by the review team. Interventions were described using the TIDieR checklist [23]. This incorporates the intervention rationale, control, intervention materials and procedure, frequency and duration, professions involved, location, and fidelity and adherence to the intervention. Fidelity was operationalised as compliance with the intervention protocol, and adherence as participant completion of the intervention. The checklist was used to describe the interventions; no scoring criteria were applied. Due to the anticipated variation, there was no formal heterogeneity assessment or data transformation.

## Results

**Fig. 1:**
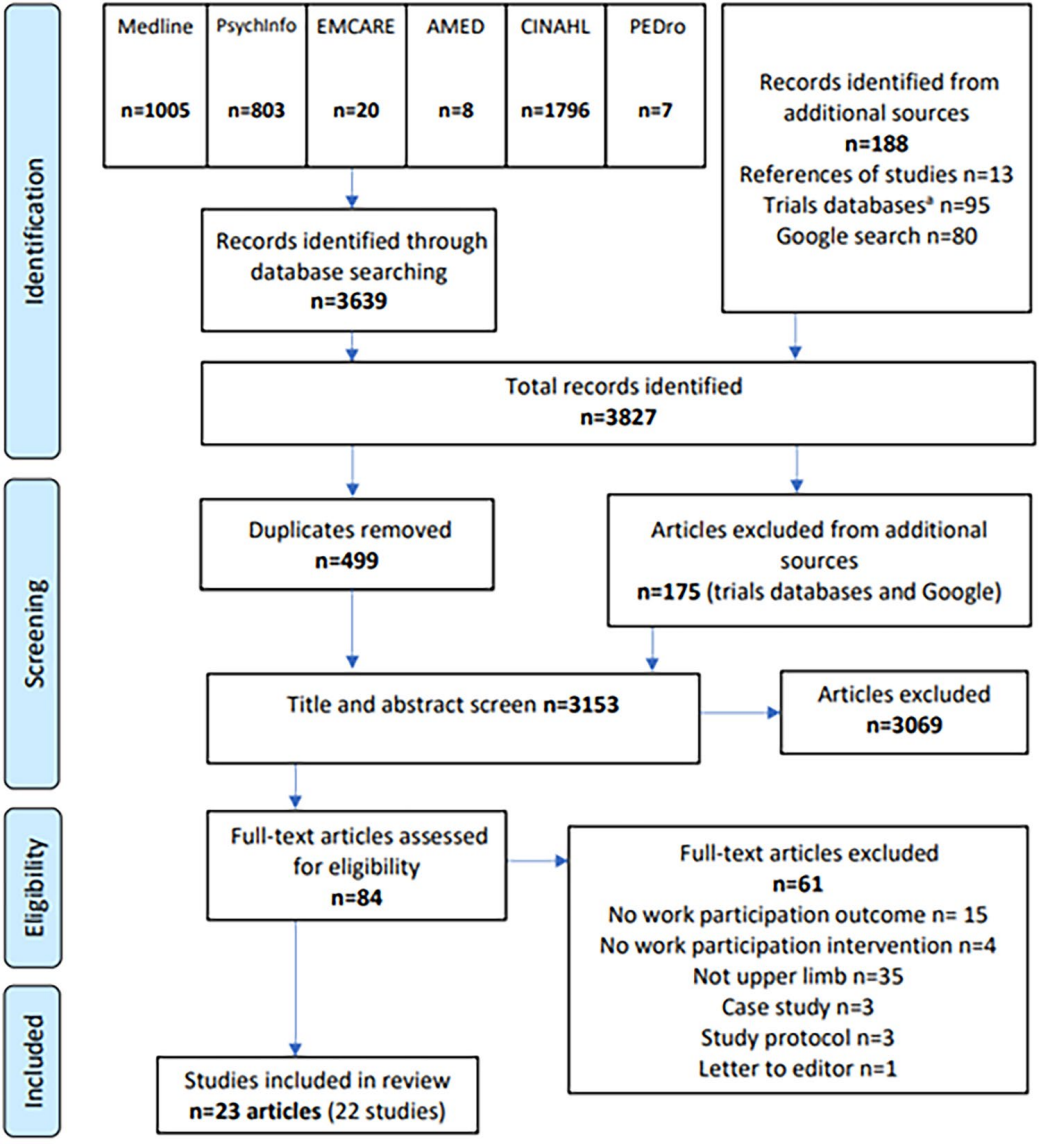
PRISMA flowchart

Twenty-two studies (23 articles) met the eligibility criteria and were included in the review from an initial yield of 3328 after removal of duplicates (Fig. 1). Articles excluded during full text screening, and reason for exclusion, are listed in Appendix 4. Six authors were contacted for additional information to support eligibility decisions and data extraction [24–29] (25/01/2024–29/02/2024); one responded [29].

### Study Characteristics

#### Design

Included articles comprised 12 randomised controlled trials (RCTs) [24–26, 30–38], four non-randomised experimental studies [39–42], five case series [29, 43–46], one mixed methods study [47], and one economic evaluation [48]. Four articles required access to other publications to identify study information, for example, protocols or reports of separate data analyses [49–52].

#### Geographical Location

Thirteen studies (14 articles) were conducted in Europe [24, 30, 32–36, 38, 39, 41, 42, 44, 47, 48], six in North America [29, 37, 40, 43, 45, 46], and three in Asia (including Turkey) [25, 26, 31]. Publication years ranged from 1993 to 2021.

#### Participants

There were 2708 included participants across all studies (Tables 2, 3, and 4). The two articles by Hutting et al. [24, 47] involved the same individuals and were only included once in this calculation. Reported mean age ranged from 33 to 51 years and the proportion of female participants ranged from 11 to 100%. Ethnicity was only reported in two studies [29, 39] (Tables 2, 3, and 4).

**Table 2:** Description of included studies: multimodal or multidisciplinary interventions

Author and study design	Study work-related focus	Intervention/control	Type of upper limb condition and method of diagnosis	Reported as work-related upper limb issue	N [providing follow-up data]^a^	Mean age in years (SD)	Gender as % female	Ethnicity (%)	Occupational context
Feuerstein 1993 USA Controlled	Long-term vocational outcomes following a multidisciplinary work rehabilitation intervention for those with chronic work-disablity with a range of upper extremity disorders	(i) Multidisciplinary work rehabilitation programme (MDT) (ii) Usual care (UC)	Upper limb tendinopathies, soft tissue injuries, peripheral neuropathies, complex regional pain syndrome, osteoarthritis of the shoulder. Diagnosed by physician evaluation and medical records review	Yes	N = 24 MDT = 19 [19] UC = 15 [15]	MDT: 38 (8.0) UC: 38 (9.7)	MDT: 74 UC: 47	NR	All receiving workers’ compensation and medical benefits
Ekberg 1994 Sweden Controlled	Magnitude of the health promoting effects of early and multidisciplinary rehabilitation	(i) Multidisciplinary rehabilitation program (MDT) (ii) Usual care (UC)	Humeral tendinitis, and other neck/shoulder issues. Diagnosed by physician	Yes	N = 107 MDT = 61 [53] UC = 46 [40]	MDT: 38 (10), range (20–54) UC: 40 (12), range (18–57)	MDT: 87 UC: 70	MDT: 34% immigrant UC: 23% immigrant	Blue collar MDT: 91%, UC: 55% Service, healthcare MDT: 9%, UC: 43% Others MDT: 0%, UC: 2% Locally, many small manufacturing companies; piecework common
Schakenraad 2004 Netherlands Case series	Effects of a multidisciplinary treatment programme on return to work in patients with chronic non-specific upper limb disorders	Multidisciplinary treatment programme	Chronic non-specific upper limb musculoskeletal disorder. Diagnosed by occupational physician	Yes	N = 41 [41]	34 (9.1)	80	NR	Visual display unit work activities All on 100% sick leave for 5 months or longer
Storrø 2004 Norway Controlled	Effects of a multidisciplinary, active intervention approach on sick leave status for patients with chronic neck and shoulder pain or low back pain	(i) Multidisciplinary intervention (MDT) (ii) Usual care (UC)	Non-specific neck and shoulder pain. Diagnosed by general practitioner	No	N = 55 MDT = 30 [30] UC = 25 [25]	MDT: 43 (10.2), range 22–63 UC: 46 (10.7), range 26–61	MDT: 77 UC: 76	NR	All with more than 6 months sick leave at start of study
Meijer 2006 Netherlands RCT	Effectiveness and cost effectiveness of a multidisciplinary treatment programme on individual and societal levels in sick-listed patients with non-specific upper extremity musculoskeletal complaints	(i) Multidisciplinary treatment programme (MDT) (ii) Usual care (UC)	Non-specific upper extremity musculoskeletal disorders. Diagnosed by occupational physician	No	N = 38 MDT = 23 [19] UC = 15 [14]	MDT: 38 (7.8) UC: 38 (9.0)	MDT: 70 UC: 67	NR	Bank or university employees
Harth 2008 Germany Controlled	Effectiveness of a patient-oriented, hand rehabilitation programme compared to a standard programme regarding return to work	(i) Patient-oriented hand rehab program (PO) (ii) standard practice (usual care, UC)	Injury to finger(s), hand and wrist, mutilating injuries, complex regional pain syndrome. Method of diagnosis NR	Yes	N = 150 PO = 75^b^ UC = 75^b^	PO: 41 (11.3) UC: 46 (9.7)	PO: 16 UC: 11	NR	The area (south-west Germany) is heavily industrial with a surrounding wine-growing region. Patients from manual professions
Shaw 2008 Canada Case series	Impacts of an onsite workplace-based program on RTW for persons with shoulder injuries, including assessment of RTW pattern	Workplace-based RTW program	Shoulder rotator cuff injuries. Method of diagnosis NR	Yes	N = 184 [184]	Range: 18–45	14 (whole organisation level)	NR	Large manufacturing plant (automotive) with ~ 4000 employees
Bernaards 2011^c^ Netherlands RCT	Cost effectiveness of a work style intervention and a work style plus physical activity intervention for computer workers with neck and upper limb symptoms	(i) Work style + physical activity (WSPA) (ii) Work style (WS) (iii) Usual care (UC)	Frequent or long-term upper limb symptoms in the preceding 6 months and/or the last 2 weeks. Diagnosed by self-report	Yes	N = 466 WS = 152 [143 cost] WSPA = 156 [144 cost] UC = 158 [146 cost]	WS: 44 (8.4) WSPA: 44 (8.5) UC: 45 (8.1)	WS: 46 WSPA: 44 UC: 38	NR	Head offices of seven Dutch companies, including insurance, science, energy, transportation policy, and taxes
Nemes 2013 Romania RCT	Efficacy of rehabilitation therapy in dentists diagnosed with common musculoskeletal disorders	(i) Rehabilitation program (rehab) (ii) medical treatment only (control)	Scapulohumeral periarthritis, cervicobrachial neuralgia, hand osteoarthritis, tendinitis/tenosynovitis of upper limb, carpal tunnel syndrome. Diagnosed by rehabilitation specialist	No	N = 240 Rehab = 145 [145] Control = 95 [95]	Rehab: 46 (10.0) Control: 45 (9.3)	Rehab = 54 Control = 58	NR	Dentists
Hutting 2015 Netherlands RCT	Effectiveness of a self-management intervention (including an eHealth module), compared with usual care, in employees with chronic non-specific complaints of the arm, neck, or shoulder	(i) Self-management intervention with E-Health module (SM) (ii) Usual care (UC)	Chronic (> 3 months) non-specific complaints of arm, neck or shoulder. Diagnosed by physiotherapist	Yes	N = 123 SM = 66 [53] UC = 57 [34]	SM: 45(11.2) UC: 48 (10.5)	SM: 83 UC: 68	NR	Two healthcare facilities n = 46 One university n = 12 General population n = 65
Hutting 2017 Netherlands Mixed methods	Experiences of participants of a self-management program for employees with complaints of the arm, neck or shoulder				N = 31 [31]	46 Range: 27–61	NR	NR	
Bean 2017 Canada Case series	Impacts of a multidisciplinary rehabilitation program on pain, disability, range of motion, strength, and work status following a work-related shoulder injury	Multidisciplinary rehabilitation program	Conservatively and surgically managed rotator cuff injuries, and impingement and tendonitis. Diagnosed by orthopaedic surgeon	Yes	N = 68 [67]	51 (10) Range: 24–68	24	NR	Mixed: Construction n = 13, manufacturing n = 11, hospitality n = 9, transport n = 11, healthcare n = 6, retail n = 6, other n = 12
Thorndal Moll 2018^d^ Denmark RCT	Impacts of a multidisciplinary intervention (MDI) compared to a brief intervention (BI) on RTW for workers on sick leave	(i) Multidisciplinary intervention (MDI) (ii)Brief intervention (BI)	Primary shoulder disorder. Diagnosed by general practitioner	Yes: 46% No: 54%	N = 21 BI = 14 [NR] MDI = 7 [NR]	BI: 42 (10.4) MDI: 40 (9.2) Range: 18–60	BI: 68 MDI: 69	NR	Workers had access to sick leave benefits for 52 weeks
Voss 2019 USA Case series	Effects of a comprehensive work rehabilitation program on RTW status, and impact of intervention timing and type of injury on RTW status at discharge	Comprehensive, interdisciplinary, work rehabilitation program	Work-related injuries of the upper quadrant, including injuries to the shoulder, elbow, forearm, wrist, hand. Diagnosed by medical practitioner	Yes	N = 246 [NR]	46 (9.8) Range: 20–66	24	African American: 20 Caucasian: 65 Hispanic: 13 Other: 2	Work level upon admission, all (n = 486): - restrictions = 153 (31.48%) - no restrictions = 2 (0.41%) - not working = 331 (68.11%)

^a^If mixed populations, only includes individuals with upper limb conditions. Completed follow-up data related to work outcomes. Loss to follow-up numbers reported for primary end point, or study end point if primary end point not reported

^b^All patients included in work-related analyses

^c^Companion paper [1]

^d^Companion paper [2]

*RCT* randomised controlled trial, *RTW* return to work. NR not reported.

**Table 3:** Description of included studies: ergonomic interventions

Author and study design	Study work-related focus	Intervention names	Type of upper limb condition and method of diagnosis	Reported as work-related upper limb issue	N [providing follow-up data]^a^	Mean age in years (SD)	Gender as % female	Occupational context
Shiri 2011^c^ Finland RCT	Effects of an ergonomic intervention on sickness absence and productivity caused by upper extremity musculoskeletal disorders	(i) Ergonomic intervention (Ergo) (ii) Usual care (UC)	Upper limb tendinopathies, nerve impingement syndromes, non-specific upper extremity pain. Consensus diagnosis by 2 specialists in physical medicine and rehabilitation	No	N = 177 Ergo = 91 [89 sickness absence, 80 productivity] UC = 86 [84 sickness absence, 76 productivity]	Ergo: 45 (10.1) UC: 46 (9.5)	Ergo: 85 UC: 89	222 expressed interest in the study: 64% healthcare workers, 25% secretaries/clerical, 8% warehouse workers
Sherrod 2014 USA Case series	Effectiveness of ergonomic and diversified chiropractic care for the reduction of impaired productivity in college workers presenting with neck and upper extremity musculoskeletal complaints	Ergonomic intervention and chiropractic care	Pain or discomfort in the upper extremity (± neck and upper back). Diagnosed by self-report	No	N = 5 [5]	Median 43 Range: 30–60	100	Staff and faculty of a chiropractic college
Esmaeilzadeh 2014 Turkey RCT	Impacts of a comprehensive and multicomponent ergonomic intervention program on computer workers, including workday loss	(i) Multicomponent ergonomic intervention program (MCIP) (ii) Control (usual care, UC)	Work-related upper extremity musculoskeletal disorders among computer workers. Diagnosed using modified Nordic Musculoskeletal Questionnaire	Yes	N = 81 MCIP = 40 [35] UC = 41 [34]	MCIP: 36 (6.5) UC: 35 (7.8)	MCIP: 74 UC: 68	University computer workers (administrators)
So 2019 Hong Kong RCT	Outcomes of an ergomotor intervention versus conventional physiotherapy on workers with work-related neck-shoulder pain and specific work demands	(i) Ergomotor intervention (EM) (ii) Conventional physiotherapy (usual care, UC)	Work-related neck-shoulder pain—defined as (average intensity during past four weeks of ≥ 2 on a 0–10 scale). Diagnosed by physiotherapist and orthopaedics	Yes	N = 101 EM = 51 [40] UC = 50 [38]	EM: 35 (8.7, range 20–49) UC: 36 (8.9, range 22–54)	CO: 48 EM: 51	Type of work: Banking & finance: EM = 6(12%), UC = 2(4%), Food/catering: EM = 1(2%), UC = 0 Engineering: EM = 1(2%), UC = 4(8%) Photographer/tourism: EM = 1(2%), UC = 2(4%) School teacher: EM = 4(8%), UC = 2(4%) Healthcare: EM = 6(12%), UC = 7(14%) Sales/retail: EM = 0, UC = 5(10%), Clerical/admin: EM = 19(37%), UC = 15(30%) IT: EM = 2(4%), UC = 3(6%) Driver: EM = 1(2%), UC = 2(4%) Academic: EM = 10(20%), UC = 8(16%)

^a^If mixed populations, only includes individuals with upper limb conditions. Completed follow-up data related to work outcomes. Loss to follow-up numbers reported for primary end point, or study end point if primary end point not reported

^b^All patients included in work-related analyses

^c^Companion paper [3]. Ethnicity not reported in any study

*RCT* randomised controlled trial, *RTW* return to work.

**Table 4:** Description of included studies: exercise interventions

Author and study design	Study work-related focus	Intervention names	Type of upper limb condition and method of diagnosis	Reported as work-related upper limb issue	N [n providing follow-up data]^a^	Mean age in years (SD)	Gender as % female	Occupational context
van den Heuvel 2003 Netherlands RCT	Effects of a software program that stimulates extra breaks and exercises on sick leave and productivity	Software program that stimulates: (i) Breaks (ii) Breaks and exercises (ii) Control (no software)	‘Repetitive strain injury’ of the neck and upper limb attributed to computer use. Diagnosed by allied occupational health physician	Yes	N = 268 Breaks = 97 [79 sick leave, 89 productivity] Breaks + Ex = 81 [65 sick leave, 69 productivity] Control = 90 [74 sick leave, 75 productivity]	Breaks: 39 Breaks + Ex: 42 Control: 37 Range: 18–50	Control: 57 Breaks: 54 Breaks + Exs:34	Single large office organisation dealing with social security allowance, across 22 locations
Cheng 2007 Hong Kong RCT	Effect of a workplace-based work hardening program on the RTW process for work-related rotator cuff disorder	(i) Workplace-based work hardening program (WWH) (ii) Clinic-based work hardening program (usual care) (CWH)	Work-related rotator cuff tendinitis. Diagnosed by medical practitioner	Yes	N = 103 WWT = 51 [46] CWH = 52 [48]	WWT: 33 (10.1) CWH: 32 (10.3)	WWT: 20 CWH: 27	Medium physical demand level of work All covered by Employees’ Compensation Ordinance
Sundstrup 2014^c^ Denmark RCT	Impact of ergonomic or strength training on work ability and disability among slaughterhouse workers with chronic pain and work disability	(i) Strength training (ii) Ergonomic training (Ergo)	Chronic (> 3 months) pain in shoulder, elbow forearm, hand/wrist. Diagnosed by self-report	No	N = 66 Strength = 33 [30] Ergo = 33 [30]	Strength: 48 (9) Ergo: 43 (9) Range: 18–67	Strength: 24 Ergo: 21	Slaughterhouse workers
Blanquero 2020 Spain RCT	Impacts of feedback-guided exercises performed on a tablet touchscreen compared with a paper-based home exercise program on RTW	(i) ReHand tablet application (ii) Evidence-based home exercise program currently used in the healthcare service (usual care)	Fractures of the hand and wrist, tendinopathies of the upper limb, soft tissue injuries of the hand and wrist, carpal tunnel syndrome, loss of digit(s), open wounds of the forearm, hand and/or finger(s). Method of diagnosis NR	Yes	N = 74 ReHand = 40 [40] UC = 34 [34]	Range: 18–65 ReHand: 45 (11) UC: 42 (11)	ReHand: 32 UC: 44	All covered by health insurance
Monticone 2021 Italy RCT	Efficacy of a rehabilitation program that incorporates task-oriented exercises and occupational therapy compared to general physiotherapy in improving disability	(i) Functional/task-oriented exercises (Task) (ii) General physiotherapy (usual care, UC)	Proximal humerus fracture (Displaced and unstable, AO type 2) managed with internal fixation. Diagnosed by orthopaedic surgeon and physiatrist	No	N = 70 Task = 35 ^b^ UC = 35 ^b^	Task: 51 (11.4) UC: 48 (11.0) Range: 23–67	Task: 63 UC: 54	Self-employed: Task = 15, UC = 13 Employed: Task = 20, UC = 22 Blue collar: Task = 18, UC = 19 White collar: Task = 17, UC = 16

^a^If mixed populations, only includes individuals with upper limb conditions. Completed follow-up data related to work outcomes. Loss to follow-up numbers reported for primary end point, or study end point if primary end point not reported

^b^All patients included in work-related analyses. c. Companion paper [4]. Ethnicity not reported in any study

*RCT* randomised controlled trial, *RTW* return to work, UC usual care.

Fifteen studies described the included upper limb conditions as work-related, although it was often unclear how this attribution was determined, for example through self-report, clinical assessment, or documented injury at work (Tables 2, 3, and 4). Five studies included populations with discrete diagnoses: rotator cuff injuries or shoulder tendinitis [25, 37, 43, 45] and proximal humeral fractures [33]. The remaining 17 studies involved mixed upper limb diagnoses including both trauma and acquired conditions. Thirteen studies included conditions of the whole upper limb [24, 29–32, 34–36, 38, 40, 44, 46, 48], seven were limited to the shoulder (± neck) [25, 26, 37, 39, 42, 43, 45], one to the proximal humerus [33], and one to the fingers, hand, and wrist [41] (Tables 2, 3, and 4).

#### Occupational Factors

Reported occupational characteristic, included workers’ compensation or other health insurance status, baseline sick leave status or work restrictions, workplace, and type of work or profession (Tables 2, 3, and 4). However, no studies reported results separately for different occupational characteristics. Specific details on national policies for occupational sick pay and related benefits were only documented by one study, which reported a national policy entitling all participants to 52 weeks of sick pay [37].

### Quality Assessment and Risk of Bias

**Fig. 2:**
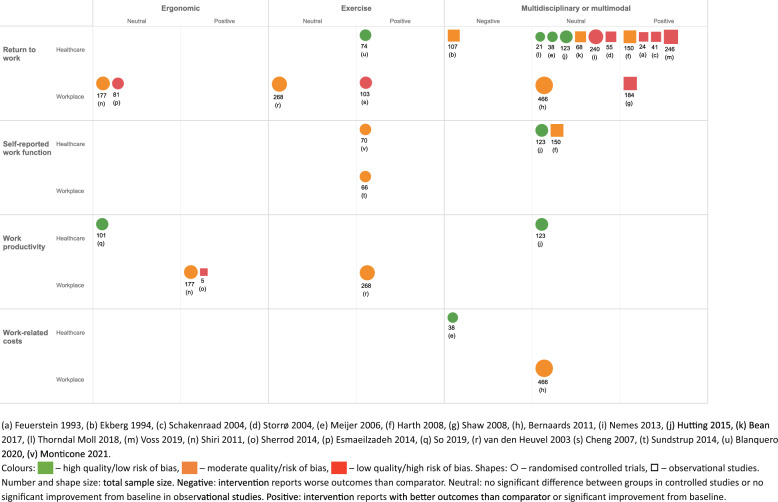
Summary of study outcomes for intervention type, setting, and outcome domain

The findings of the methodological quality assessment are summarised in Fig. 2 and presented in full in Appendix 5. Five studies (23%) were rated as high quality. None of the RCTs reported participant or intervention-delivery blinding due to the nature of the intervention. There were no placebo-controlled trials; control groups were often ‘usual care’, which was not comprehensively described. Two RCTs did not report sample size calculations [31, 34] and four reported issues with under-recruitment or loss to follow-up [33–35, 37]. This was also an issue for two quasi-experimental studies [40, 42]. Three case series were assessed as missing key demographic and clinical information, including occupational information for participants, method of diagnosis or duration of the upper limb condition, or previous treatment [29, 44, 45]. Both the mixed method study [47] and economic evaluation [48] scored highly on their respective quality appraisal tools.

### Work Participation Outcomes

There were four reported outcome domains: return to work, self-reported work functioning, work productivity, and work-related costs. There was heterogeneity in both the specific outcome measures used within each domain and the timing of data collection (Tables 5, 6, and 7).

**Table 5:** Reported outcomes for multimodal and multidisciplinary interventions

Article	Intervention	Outcome measure	Baseline	Time 1 (≤ 3 months)	Time 2 (> 3 months ≤ 12 months)	Time 3 (> 12 months, ≤ 24 months)	Clinical relevance
Feuerstein 1993 USA*	Multidisciplinary work rehabilitation programme (MDT) vs usual care (UC)	Work status: proportion (i) RTW (ii) In vocational training	0	–	–	(i) RTW MDT: 58% UC: 40% (ii) Vocational training MDT: 20% UC: 0%	Larger proportion RTW or training in MDT group (descriptive statistics only)
Ekberg 1994 Sweden**	Multidisciplinary rehabilitation program (MDT) vs usual care (UC)	Sick leave due to MSK disorder: mean days [95% CIs]	Preceding 12 months MDT: 28 [19, 37] UC: 25 [14, 34]	MDT: 72 [68, 86] UC: 48 [40, 56]	6 months MDT: 36 [28, 45] UC: 21 [11, 30] 9 months MDT: 24 [15, 31] UC: 20 [11, 29]	MDT: 11 [4, 17] UC: 14 [5, 23]	Statistically significant difference in sick leave in first 12 months with less sick leave in UC group. NSD after 24 months
		Work status: proportion RTW	MDT: 3 (6%) UC: 3 (8%)	MDT: 21% UC: 63%	MDT: > 80% UC: > 80%	–	
Schakenraad 2004 Netherlands*	Multidisciplinary treatment programme	Work status: proportion RTW (i) Full time (ii) Part time (iii) Adjusted hours	0	–	(i) Full time: 26 (63%) (ii) Part time: 9 (22%) (iii) Adjusted hours: 6 (15%)	–	63% returned to usual work duties and hours
Storrø 2004 Norway*	Multidisciplinary intervention (MDT) vs usual care (UC)	Work status: proportion RTW	MDT: 0 UC: 0	1 month MDT: 18 (60%) UC: 12 (48%) 3 months MDT: 22 (73%) UC: 13 (52%)	MDT: 24 (80%) UC: 14 (56%)	MDT: 25 (83%) UC: 15 (60%)	NSD between groups
Meijer 2006 Netherlands***	Multidisciplinary treatment programme (MDT) vs usual care (UC)	Work status: proportion RTW [95% CIs] 100% = total return to usual work duties and hours	MDT: 28.5% [15.0,42.0] UC: 29.2% [13.4, 44.9]	MDT: 39.6% [25.1, 54.1] UC: 38.1% [21.3, 55.0]	MDT: 81.9% [65.4, 98.3] UC: 71.6% [52.5, 90.8]	MDT: 86.0% [68.5, 103.4] UC: 72.8% [52.5, 93.2]	NSD between groups
		Costs including direct medical and nonmedical (mean [95% CIs]): (i) Total costs (ii) Cost of productivity losses	(i) Total costs MDT: €495 [402, 588] UC: €429 [3223, 541] (ii) Production loss MDT: €391 [NR] UC: €363 [NR]	(i) Total costs MDT: €1336 [1247, 1425] UC: €448 [345, 551] (ii) Production loss MDT: €366 [NR] UC: €344 [NR]	(i) Total costs MDT: €665 [589, 740] UC: €359 [272, 447] (ii) Production loss MDT: €277 [NR] UC: €273 [NR]	(i) Total costs MDT: €431 [361, 501] UC: €316 [234, 397] (ii) Production loss MDT: €219 [NR] UC: €239 [NR]	Statistically significant lower costs with usual care at 2 and 6 months, NSD at 12 months
Harth 2008 Germany**	Patient-oriented hand rehab programme (PO) vs usual care (UC)	Sick leave: days between start of rehabilitation and RTW	0 (all off work)	–	PO: 92.3 UC: 191.3	–	Earlier RTW in PO group (descriptive statistics only). Suggests a cost saving in sickness benefits
		Work status: proportion returned to original job	0	–	PO: 33.9% UC: 12.3%	–	
		DASH Work (mean)	PO: 62.5 UC: 93.5	–	PO: 26.0 UC: 59.8	–	NSD between groups
Shaw 2008 Canada*	Workplace-based RTW program	Sick leave: cumulative proportion returned to pre-injury level of work or working with restrictions (i) Regular duties (ii) Modified duties	0	(i) Regular duties By 7 days: 24 (13.2%) By 14 days: 43 (23.6%) By 29 days: 77 (41.7%) (ii) Modified duties By 3 days: 61 (33.0%) By 7 days: 86 (47.3%) By 14 days: 107 (58.2%) By 30 days: 136 (73.6%)	(i) Regular duties By 58 days: 115 (62.5%) By 88 days: 138 (75%) By 120 days: 155 (84%) (ii) Modified duties By 63 days: 155 (84.1%)	–	87.8% returned to regular duties by the end of the study period
Bernaards 2011 Netherlands**	Work style + physical activity (WSPA) vs Work style (WS) vs Usual care (UC)	Sick leave: mean days [SD]	Preceding 12 months WS: 15.0 [39.7] WSPA: 21.1 [53.9] UC: 13.0 [35.0]	–	–	WS: 14.1 [33.2] WSPA: 20.5 [52.3] UC: 17.6 [36.0]	Mean difference [95% CI] WS: − 0.98 [− 8.4, 6.4] WSPA: − 0.92 [− 11.1, 9.2] UC: 4.67 [− 2.8, 12.1]
		Sick leave: mean number of episodes [SD]	WS: 1.7 [1.9] WSPA: 1.9 [2.2] UC: 1.7 [1.9]	–	–	WS: 1.9 [2.3] WSPA: 2.0 [2.0] UC: 2.0 [2.1]	Mean difference [95% CI] WS: 0.27 [− 0.07, 0.60] WSPA: 0.10 [− 0.24, 0.44] UC: 0.31 [0.03, 0.59]
		Sick leave: 12-month prevalence (> = 1 day)	WS: 66.4% WSPA: 70.1% UC: 67.8%	–	–	WS: 69.9% WSPA: 74.3% UC: 73.3%	Mean difference [95% CI] WS: 3.5 [− 4.5, 12.9] WSPA: 4.2 (− 4.9, 11.9) UC: 5.5 (− 1.9, 12.9)
		Cost to employer (mean [SD]): (i) Intervention costs (ii) Productivity losses (iii) Sickness absence	NR	–	–	(i) Intervention costs WS: €108 [6.5] WSPA: € 141 [6.2] UC: €16 [6.3] (ii) Productivity loss WS: €1799 [3851] WSPA: €2670 [6356] UC: €2294 [4564] (iii) Sickness absenteeism: WS: €1758 [3855] WSPA: €2616 [6358] UC: €2294 [4565]	NSD in cost effectiveness between groups. Difference in costs [95% CIs] (i) Intervention costs: WS vs UC: €92 [90, 93] WSPA vs UC: €125 [124, 127] WSPA vs WS: €33 [32, 35] (ii) Productivity loss: WS vs UC: € − 543 [− 1499, 414] WSPA vs UC: €104 [− 1136, 1344] WSPA vs WS: €661 [− 517, 1839] (iii) Sickness absence: WS vs UC: € − 583 [− 1541, 374] WSPA vs UC: €51 [− 1190, 1291] WSPA vs UC: €648 [− 531, 1826]
Nemes 2013 Romania*	Rehabilitation program (Rehab) vs medical treatment only (Control)	Sick leave: mean days [SD]	Rehab: 6.77 [0.89] Control: 6.47 [0.90]	–	–	Rehab: 3.45 [1.07] Control: 5.61 [0.76]	Statistically significant improvement in both groups
Hutting 2015 Netherlands***	Self-management intervention with E-Health module (SM) vs usual care (UC)	Sick leave: mean days absent in past month	SM: 1.63 UC: 3.70	SM: 4.21 UC: 4.02	SM: 2.45 UC: 7.84	SM: 3.42 UC: 7.27	NSD between groups
		Upper limb-related sick leave: mean days absent in past month	SM: 0.81 UC: 3.64	SM: 0.74 UC: 2.62	SM: 0.38 UC: 3.51	SM: 1.08 UC: 5.27	NSD between groups
		DASH Work (mean)	SM: 28.8 UC: 30.2	SM: 19.6 UC: 22.2	SM: 15.7 UC: 20.8	SM: 12.9 UC: 17.6	Significant difference in favour of SM. Mean difference [95% CIs] − 3.82 [− 7.46, − 0.19]
		Stanford Presenteeism scale: score 6–30, 30 = higher presenteeism (mean)	SG = M: 22.2 UC: 22.1	SM: 23.0 UC: 23.0	SM: 23.3 UC: 22.3	SM: 23.7 UC: 23.3	NSD between groups. Mean difference (95% CIs] 0.12 [− 0.81, 1.05]
		Work Limitations Questionnaire: score range 0–100, 0 = no limitations (mean)	SM: 7.2 UC: 7.7	SM: 7.7 UC: 7.7	SM: 7.5 UC: 7.0	SM: 6.1 UC: 7.5	NSD between groups. Mean difference 95% CIs] 0.07 [− 0.70, 0.84]
		Questionnaire on the Experience and evaluation of work subscales: lower score = better outcome. Score range NR (mean) (i) Pace and amount of work (ii) Participation in work	(i) Pace and amount of work SM = 27.72 UC = 29.08 (ii) Participation in work SM = 53.51 UC = 50.44	(i) Pace and amount of work SM = 27.90 UC = 26.01 (ii) Participation in work SM = 52.44 UC = 50.29	(i) Pace and amount of work SM = 26.56 UC = 25.04 (ii) Participation in work SM = 49.21 UC = 52.59	(i) Pace and amount of work SM = 37.73 UC = 42.86 (ii) Participation in work SM = 50.48 UC = 49.75	NSD between groups. Mean difference [95% CIs] (i) Pace and amount of work 0.22 [0.31, 0.75] (ii) Participation in work − 0.49 [− 3.21, 2.24]
		Work limitation in previous 2 weeks: score 0–10, 0 = no limitations (mean)	SM: 3.5 UC: 4.0	–	–	SM: 2.1 UC: 3.1	Significant difference in favour of SM. Mean difference [95% CIs]: − 1.01 [− 1.97, − 0.04]
		Work capacity: score 0–10, 0 = no limitations (mean)	SM: 7.3 UC: 6.7	–	–	SM: 7.6 UC: 7.3	NSD between groups. Mean difference (95% CIs]: − 0.04 [− 0.89, 0.81]
		Self-efficacy and at work scale: higher score = better outcome (mean). Score range NR	SM: 8.6 UC: 6.2	SM: 15.1 UC: 9.6	SM: 11.5 UC: 9.2	SM: 13.6 UC: 9.4	NSD between groups. Mean difference [95% CIs]: 0.11 [− 0.49, 5.14]
		Workstyle short form: mean score Higher score = worse score, ≥ 28 = adverse score	SM: 33.05 UC: 33.85	SM: 28.96 UC: 31.81	SM: 27.02 UC: 33.27	SM: 27.64 UC: 30.61	Overall treatment effect, adjusted for confounders (duration of symptoms, gender, age): − 1.28 [95% CIs − 4.16, 1.59]. NSD between groups
Bean 2017 Canada*	Multidisciplinary rehabilitation program	Work status: (i) full time work (ii) part time work (iii) off work	(i) Full time: 18 (26%) (ii) Part time: 15 (22%) (iii) Off work: 35 (51%)	–	–	(i) Full time: 40 (59%)^a^ (ii) Part time: 11 (16%)^a^ (iii) Off work: 16 (24%)^a^	Change in work status: Improved (progressed from off work to at work or from part time to full time): 29 (43%) No change: 36 (53%) Worse: 1 (1%)
Thorndal Moll 2018 Denmark***	Multidisciplinary intervention (MDT) vs Brief intervention (BI)	Sick leave: weeks sickness absence (median [IQR]) From national benefits registry	< = 12 weeks sick leave duration MDT: 66 (78%) BI: 60 (72.3)	–	–	MDT: 44 [18–52] BI: 32 [12–52]	NSD between groups
		Work status at 12 months: proportion in work	0	–	–	MDT: 50 (59%) BI: 48 (58%)	
Voss 2019 USA*	Comprehensive, Interdisciplinary, work rehabilitation Program	Work status: proportion achieving i–iii (i) new occupation capacity (ii) work with restrictions (iii) work with no restrictions (iv) not working	85 (35%)	–	–	205 (84%)^a^ On discharge	Nearly 3 times as many individuals in work at the end of the study period

^a^Timepoint not reported

*Low quality/high risk of bias

**Moderate quality/moderate risk of bias

***High quality/low risk of bias

*NSD* no significant difference, *RTW* return to work, *SD* standard deviation, *IQR* interquartile range, *CI* confidence intervals.

**Table 6:** Reported outcomes for ergonomic interventions

Article	Intervention	Outcome measure	Baseline	Time 1 (≤ 3 months)	Time 2 (> 3 months ≤ 12 months)	Time 3 (> 12 months, ≤ 24 months)	Clinical relevance
Shiri 2011 Finland**	Ergonomic intervention (Ergo) vs Usual care (UC)	Sick leave: mean days [SD] from company records		Ergo: 6.2 [3.3] UC: 9.8 [10.2]	Ergo: 23.3 [23.6] UC: 17.3 [17.3]	–	NSD between groups
		Sick leave: proportion off work	Ergo: 35.7% UC: 38.1%	Ergo: 10.1% UC: 11.9%	Ergo: 10.1% UC: 16.7%	Ergo: 19.1% UC: 23.8%	NSD between groups
		Self-assessed productivity loss at work: (i) Proportion experiencing any productivity loss (%) (ii) Magnitude (mean reduction in productivity, [SD], scored 0–100)	Ergo (i) Proportion: 45 (53.8%) (ii) Magnitude: 16.7 [SD NR] UC (i) Proportion: 49 (57.9%) (ii) Magnitude: 19.8 [SD NR]	Ergo: (i) Proportion: 24 (32.9%) (ii) Magnitude:10.9 [21.2] UC: (i) Proportion: 30 (44.1%) (ii) Magnitude = 13.8 [22.2]	Ergo (i) Proportion: 20 (25.0%) (ii) Magnitude: 6.8 [17.4] UC (n = 76) (i) Proportion: 39 (51.3%) (ii) Magnitude: 18.4 [25.7]	–	NSD between groups at 8 weeks Statistically significant improvement in both proportion and magnitude in favour of Ergo at 12 weeks
Sherrod 2014 USA*	Ergonomic intervention + chiropractic care	Self-reported percentage work productivity (median [IQR])	85 [72.5–90]	100 [95–100]	16 weeks: 100 [80–100] 52 weeks: 100 [96.25–100]	–	Improvement in productivity after intervention, maintained at 12 months
Esmaeilzadeh 2014 Turkey*	Multicomponent ergonomic intervention program (Ergo) vs usual care (UC)	Sick leave in last 3 months due to work-related upper extremity musculoskeletal symptoms, self-reported as: 1 = Never 2 = 1–7 days 3 = 8–14 days 4 = 15–28 days 5 = > 1-month	Median [range] Ergo: 1.0 [1–2] UC: 1.0 [1–2]	–	Change in sick leave score. Median [range] Ergo: 0.0 [− 0.5 to 1.0] UC: 0.0 [− 0.5 to 1.0]	–	NSD between groups
So 2019 Hong Kong***	Ergomotor intervention (EM) vs Usual care (UC)	Chinese version of Workstyle short form: mean score (SD) Higher score = worse score, ≥ 28 = adverse score	EM: 45.4 [13.1] UC: 45.5 [14.3]	EM: 36.9 [15] UC: 40.2 [12.9]	–	–	NSD between groups

*Low quality/high risk of bias

**Moderate quality/moderate risk of bias

***High quality/low risk of bias

*NSD* no significant difference, *SD* standard deviation, *IQR* interquartile range.

**Table 7:** Reported outcomes for exercise interventions

Article	Intervention	Outcome measure	Baseline	Time 1 (≤ 3 months)	Time 2 (> 3 months ≤ 12 months)	Time 3 (> 12 months, ≤ 24 months)	Clinical relevance
van den Heuvel 2003 Netherlands**	Software program that stimulates Breaks and exercises vs Breaks only vs Control	Sick leave: proportion off work	Breaks + Ex: 7 (10.8%) Breaks: 5 (6.3%) Control: 7 (9.5%)	Breaks + Ex: 4 (6.2%) Breaks: 3 (3.8%) Control: 4 (5.4%)	–	–	NSD between groups
		Productivity: accuracy rate measured as: 1—(backspace + delete)/Total keystrokes	NR	Breaks + Ex: 95 Breaks: 95 Control: 93	–	–	Statistically significant difference in favour of both interventions compared with the control group
Cheng 2007 Hong Kong*	Workplace-based work hardening program (WWH) vs clinic-based work hardening program (CWH)	Work status: proportion RTW (i) Normal duties (ii) Modified duties	All participants off work	WWH: 33 (71.7%) (i) Normal duties: 21 (45.7%) (ii) Modified duties: 12 (26.1%) CWH: 18 (37.5%) (i) Normal duties 16 (33.3%) (ii) Modified duties:2 (4.2%)	–	–	Significant difference in favour of WWH for return to work at 4 weeks
Sundstrup 2014 Denmark**	Strength training vs ergonomic training (usual care, UC)	Work ability index (WAI): score 7–49: 7–27 = poor work ability 28–36 = moderate work ability 37–43 = good work ability 44–49 = excellent work ability. Mean (SD). Total score and component items: (i) Current work ability compared with lifetime best (0–10) (ii) Work ability in relation to job demands (2–10) (iii) Number of current physician-diagnosed diseases (1–7) (iv) Estimated work impairment due to disease (1–6) (v) Sick leave during past year (1–5) (vi) Own prognosis of work ability 2 years from now (1–7) (vii) Mental resources (1–4)	WAI Strength: 39.2 [3] Ergonomic: 39.4 [3] (i) Current work ability Strength: 7.3 [1] Ergonomic: 7.2 [1] (ii) Work ability vs job demands Strength: 7.5 [0.9] Ergonomic: 7.5 [0.9] (iii) Diagnosed diseases Strength: 5.6 [0.1] Ergonomic = 5.6 (0.9) (iv) Work impairment Strength: 5.7 [0.4] Ergonomic: 5.7 [0.4] (v) Sick leave Strength: 4.7 [0.6] Ergonomic: 4.6 [0.6] (vi) Prognosis of work ability Strength: 5.5 [0.4] Ergonomic: 5.7 [0.4] (vii) Mental resources Strength: 3.0 [0.5] Ergonomic: 3.0 [0.5]	Mean change from baseline [95% CIs] WAI Strength: 0.3 [− 1.1, 1.7] Ergonomic: − 2.2 [− 3.5, − 0.8] (i) Current work ability Strength: 0.0 [− 0.5, 0.5] Ergonomic: − 0.5 [− 0.9, 0.0] (ii) Work ability vs job demands Strength: 0.4 [0.0, 0.8] Ergonomic: − 0.3 [− 0.8, 0.1] (iii) Diagnosed diseases Strength: − 0.2 [− 0.6, 0.3] Ergonomic = − 0.3 (− 0.7 to 0.1) (iv) Work impairment Strength: 0.1 [− 0.1, 0.3] Ergonomic: 0.0 [− 0.2, 0.2] (v) Sick leave Strength: − 0.2 [− 0.6, 0.0] Ergonomic: − 0.5 [− 0.8, − 0.2] (vi) Prognosis of work ability Strength: 0.2 [− 0.5, 0.8] Ergonomic = − 0.3 (− 0.9 to 0.3) (vii) Mental resources Strength: 0.1 [− 0.1, 0.4] Ergonomic: − 0.3 [− 0.5, 0.0]	–	–	Between group difference mean [95% CIs] WAI—significant difference in favour of Strength 2.3 [0.9 to 3.7] (i) Current work ability—NSD 0.5 [0.0, 1.0] (ii) Work ability vs job demands—significant difference in favour of Strength 0.7 [0.3, 1.2] (iii) Diagnosed diseases—NSD 0.1 [− 0.3, 0.6] (iv) Work impairment—NSD 0.0 [− 0.2, 0.2] (v) Sick leave—NSD 0.2 [− 0.1, 0.5] (vi) Prognosis of work ability—NSD 0.3 [− 0.4, 1.0] (vii) Mental resources—significant difference in favour of Strength 0.3 [0.1, 0.6]
		DASH Work: score 0–100, 0 = no restrictions to work function (mean [SD])	Strength: 28.3 [13.8] Ergonomic: 27.8 [13.8]	Mean change from baseline [95% CIs) Strength: − 6.5 [− 13.2, 0.1] Ergonomic: 2.8 [− 3.7, 9.4]	–	–	Significant difference in favour of Strength. Between group difference mean [95% CI] − 8.8 [− 15.6, − 2.0]
Blanquero 2020 Spain***	ReHand tablet application vs evidence-based home exercise programme provided on paper (Usual care UC)	Sick leave: days sickness absence (mean [SD]) Calendar days between first day of sick leave and discharge from health insurance company to RTW	Baseline sickness absence not reported	ReHand: 76 [SD 33] UC: 94 [SD 32]	–	–	Difference of 7 days considered smallest effect to outweigh additional cost of ReHand application. Between group difference of 18 days in favour of ReHAnd (95% CI − 33, − 3)
Monticone 2021 Italy**	Functional/task-oriented exercises (Task) vs usual care (UC)	DASH Work: mean [SD; 95% CIs]	Task: 78.8 [23.0; 70.85, 86.65] UC: 78.0 [24.0; 69.80, 86.28]	Task: 50.4 [26.2; 41.08, 59.68] UC: 68.6 [24.9; 59.87, 77.26]	Task: 36.9 [27.1; 27.14, 46.68] UCS: 56.7 [26.4; 46.98, 66.32]	–	Significant difference in favour of Task. Mean difference [95% CIs]: 3 months − 18.19 [7.80, 28.57] 12 months − 19.74 [9.03, 30.45]

*Low quality/high risk of bias

**Moderate quality/moderate risk of bias

***High quality/low risk of bias

*NSD* no significant difference, *RTW* return to work, *SD* standard deviation, *CI* confidence intervals.

### Work Participation Interventions

Interventions were categorised into three groups: (i) multidisciplinary or multimodal, (ii) ergonomic, and (iii) exercise. The first comprised mixed interventions delivered by more than one professional group and incorporating physical and psychological aspects. The second focussed on the design, layout, or interaction with the work environment. The third used prescribed exercise or activity programmes with a focus on building strength, flexibility, and/or endurance. Interventions were described using the TIDieR checklist [23] and categorised before the study findings were explored to prevent potential bias in the allocation (Appendix 6).

Where control groups were included in the study design, the control was most frequently described as usual care, although the composition of ‘usual care’ was not always defined. Control group interventions included physiotherapy [26, 30, 33, 39, 40, 42, 48], medical interventions [32, 37, 39, 40, 48], information provision [38], and work hardening [25, 36] (Appendix 6).

Where reported, mean age was similar across all intervention categories. Age ranged from 34 to 51 years for studies evaluating multidisciplinary or multimodal interventions, 35–46 years for studies evaluating ergonomic interventions, and 32–51 years for studies evaluating exercise interventions (Tables 2, 3, and 4). Gender distributions were more variable, both between groups in individual studies and across intervention categories (Tables 2, 3, and 4). Where reported, > 60% of participants were female in seven of the multidisciplinary or multimodal intervention groups (54%), three of the ergonomic interventions (75%), and one of the exercise interventions (20%) (Tables 2, 3, and 4).

Eight interventions were primarily delivered in the workplace [25, 31, 35, 36, 38, 45, 46, 48], compared with 14 in healthcare settings [24, 26, 29, 30, 32–34, 37, 39–44]. Summary outcomes for each intervention category are presented in Fig. 2.

### Characteristics and Effectiveness of Multidisciplinary or Multimodal Interventions

The 13 interventions categorised as multidisciplinary (MDT) or multimodal interventions all involved combined physical and psychological rehabilitation, delivered in a variety of formats, including group sessions, 1:1 sessions, and self-directed online programmes [24, 29, 32, 34, 37, 39–45, 47, 48] (Table 2, Appendix 6). Psychological components included stress and pain management strategies [24, 32, 40–42, 44, 47, 48], vocational counselling and work goal setting [24, 29, 37, 40, 41, 43–45, 47, 48], general health promotion, [24, 39, 47] and supported return to work [32, 34, 37, 39, 41–43, 45, 48]. Physical components included home exercises programmes [34, 40], 1:1 or group physical training [29, 32, 39, 42, 43, 48], sporting activities, e.g. bowling [32, 41], and workplace ergonomic evaluations [29, 40, 45, 48].

Twelve studies reported intervention dose. Frequency ranged from daily to monthly and duration ranged from 3 weeks to 2 years. Intervention fidelity and reported adherence were good in the three studies where both were recorded [32, 45, 48] (Appendix 6).

Nine studies included at least one comparator group, and of these three reported some benefits of the intervention compared to control for at least one outcome (Table 5). Feuerstein et al. reported larger proportion of participants returning to work or training in the MDT intervention group compared with usual care [40], and Harth et al. reported earlier return to work in the patient-oriented rehabilitation programme compared to usual care [41]. Hutting et al. reported improvements in two outcomes from a series of assessments: Disabilities of the Arm, Shoulder and Hand (DASH) work scores, and self-reported work limitations at 12 months were significantly better in the group receiving e-health and self-management interventions compared with usual care. However, they found no significant difference in other measures of return to work, self-reported work function, or productivity [24], leading to an overall assessment of no discernible difference between groups (Table 5).

Two studies reported negative outcomes associated with the intervention. Ekberg et al. found a statistically significant increase in sick leave in the MDT intervention group in the first 12 months compared with usual care [39]. This was attributed to time off work to participate in the intervention. There was no significant difference at 24 months. Meijer et al. reported significantly increased healthcare costs for the MDT intervention compared with usual care, but found no significant difference in work productivity costs, nor the proportion of participants who returned to work at each time point [32]. Healthcare costs comprised the cost of the MDT intervention (13 full days of workshops, 1:1 sessions, and sporting activities) compared with usual care coordinated by an occupational physician. Differential healthcare costs were not specifically reported in the other studies and are not considered a primary outcome of our review.

The remaining studies reported no significant differences in outcomes between intervention and control groups [34, 37, 42, 48].

Three studies without comparators reported beneficial outcomes of the intervention on return to work outcomes [29, 43–45]; one also reported a neutral finding that > 50% of participants had no change in their work status after the intervention [43].

### Characteristics and Effectiveness of Ergonomic Interventions

Four studies assessed ergonomic interventions, including activity modification and workplace assessment and adaptations [26, 31, 35, 46] (Table 3, Appendix 6). No studies reported both intervention fidelity and adherence. Only three studies reported intervention frequency and duration: 16 sessions over 12 weeks [26], weekly over 16 weeks [46], and two 90-min group sessions [31].

Three studies included comparator (usual care) groups [26, 31, 35]. The only significant difference was reported by Shiri et al. at 12 weeks: improved self-assessed productivity in the ergonomic intervention compared to usual care [35]. Sherrod et al. did not include a comparator group, and reported improved productivity following ergonomic and chiropractic interventions, maintained at 12 months [46] (Table 6).

### Characteristics and Effectiveness of Exercise Interventions

Five studies (all RCTs) assessed exercise interventions, including workplace software to prompt breaks and exercise [38], strengthening programmes [25, 36], task-orientated activities [33], and tablet-based hand exercises [30] (Table 4, Appendix 6).

Intervention dose ranged from twice a day for 4 weeks to three times per week for 12 weeks. Intervention fidelity and adherence were reported by one study [33].

All studies reported significant differences in favour of the intervention (Table 7). These were improved productivity [38], greater return to work at 4 weeks [25], reduced sick leave [30], and improved self-reported work function [33, 36]. One study found no significant difference in sick leave [38].

## Discussion

This systematic review identified 22 studies that evaluated work participation interventions for adults with upper limb MSDs. Interventions were categorised into three types, multidisciplinary or multimodal, ergonomic, and exercise. While formal meta-analysis was not possible due to variation in outcome type and timescale, this review found that exercise interventions may have a positive impact on return to work, self-reported work function, and work productivity for adults with MSDs. Findings were mixed for the other intervention categories. Importantly, two studies reported negative outcomes associated with multidisciplinary or multimodal interventions. These related to increased sick leave in the intervention group and increase costs. Clinical, demographic, and occupational contextual factors were infrequently reported, and it was therefore not possible to identify whether intervention effectiveness varied for different subgroups.

All of the studies exploring exercise were RCTs and focussed on upper limb conditioning activities to improve work ability. The exercise programmes were functional, rather than targeting individual muscles or muscle groups, although the nature of the exercise content and delivery varied; exercises presented during screen breaks without additional equipment [38], supervised strengthening with or without additional equipment [25, 33, 36]; and home exercises with a tablet [30]. Functional strengthening programmes are commonly designed to enhance muscle strength, endurance, and coordination. The combination of these elements could lead to improved work outcomes by facilitating confidence in the participant’s physical capabilities. Reported adherence to the exercise programmes ranged from 80 to 95% (Appendix 6), higher than general estimates of 50% [53]. Recommendations of who might benefit the most from these exercise interventions and in what setting is not possible from the available data. Most studies included in the review reported complex interventions (for both intervention and control groups), that is there were multiple components, often targeted at behaviour change, that required expertise to deliver and were delivered by multiple individuals in different settings [54]. Contextual factors may therefore play a key role in establishing the effectiveness and broader impacts of these work participation interventions [54]. From the information available, this review was able to consider five study design and demographic contextual factors.

For study design, positive outcomes of the intervention were less common in studies rated at low risk of bias/high quality (one out of 12 positive outcomes), while there was an even split between RCTs and observational studies.

For demographic factors, there was no observed pattern between the geographical location of the study and reported positive intervention effect. Similarly, there was no observed pattern between studies labelling the diagnosis as work-related compared with those who did not, or interventions delivered in the workplace versus healthcare settings. It was not possible to explore the impact of workers’ compensation, sick leave benefits, or type of work because these were inconsistently reported. We acknowledge that this information would enable greater understanding of the contextual factors involved and encourage healthcare researchers to collect and present a summary of this information when reporting future studies.

It was not possible to separate outcomes for other demographic factors, including age, sex, and ethnicity. Future research should report subgroup information to support exploration of potential interactions between demographic factors and health and work outcomes [55, 56].

Upper limb MSDs that are associated with work functioning are often labelled work-related upper limb conditions. This was the case for more than two-thirds of the studies included in this review. However, the causal aetiologies are often not clear cut, and it has been suggested that work-relevant is a more appropriate terminology [57]. Furthermore, a work-related diagnostic label may have a potential negative influence on occupational outcomes. Diagnostic labels have been associated with negative consequences for patients including increased psychological distress, preference for invasive treatments, and increased sick role behaviours [58]. In the current review, there was no identifiable pattern between the label of work-related and reported intervention effectiveness, but we suggest the adoption of ‘work-relevant’ (rather than work-related) terminology in both clinical practice and research, unless the aetiology is clear.

Many of the interventions were resource intensive in terms of the frequency and duration of the intervention and the clinicians or other professionals involved, however, only two studies explored economic outcomes. Future research should incorporate healthcare economic data with transparent discussions around acceptable cost–benefit profiles based on injury severity and different work-related outcomes across health and work settings. Environmental sustainability should also be considered [59]. None of the studies included assessments of the intervention/control carbon footprint or other measures of environmental sustainability and this should be incorporated into future research [60].

Many of the included studies used patient-reported outcomes, which are a key component of both patient-centred care and healthcare research [61]. For work participation interventions, it is often not possible to blind participants to intervention allocation, but studies could incorporate clearly defined ‘usual care’ interventions that also represent a change from existing practice. The perceived impact of participant expectations, particularly on self-reported measures, should be explored and reported as part of future studies [62]. Qualitative data from the included study by Hutting et al. identified perceived benefits of the study intervention on knowledge of the upper limb condition, behaviour change, and improved coping mechanisms [47], but this did not equate to a statistically significant benefit of the intervention.

Some studies incorporated the use of national databases for work-related data, including duration of sick leave. A core outcome set for work and health research is in development [63] and should be used to inform future research in this area.

None of the included studies took place within the UK National Health Service and none evaluated the impact of Fit Notes or other specific return to work documentation on work participation outcomes. Given the recent change to UK legislation to enable physiotherapists and occupational therapists to issue Fit Notes [14], this warrants targeted research.

### Strengths and Limitations

This review employed a sensitive search strategy, including hand searching and inclusion of a broad range of study types, not solely RCTs. Upper limb functioning has a unique role in work participation, and the focus on upper limb MSDs adds to the knowledgebase for physiotherapists, occupational therapists, and other healthcare professionals delivering upper quadrant rehabilitation.

The three intervention classification groups were developed as part of the review process using the data extracted for the TiDieR checklist. In the absence of a standardised classification system, interventions were grouped according to the key components outlined in the study text and the intervention name used by the study authors. Grouping and definitions were agreed by the review team, including three clinical hand therapists, and the classification was considered relevant for clinical practice. We appreciate that alternative classification strategies could have been adopted.

Similarly, outcome domains were defined by the review team. As discussed above, a work and health core outcome set is in development and we suggest that is used to guide future research and reviews in this area.

The review was limited to English language due to funding constraints. However, the included databases did present English translations of abstracts in other languages, and none met the eligibility for full text screening. It is still possible that relevant articles published in other languages were missed. Interestingly, no UK studies met the review inclusion criteria. In addition, it is not possible to exclude the impact of potential publication bias on the articles available for our review.

This review included mixed upper limb diagnoses, rather than a specific group of patients (for example following wrist fracture). This represents the mixed caseloads seen in clinical practice and the role of the whole upper quadrant in work participation. It was beyond the scope of this review to compare interventions and outcomes for adults with upper limb trauma versus acquired conditions. However, different patterns of onset may have different impacts on work participation for the individual and be associated with different types of interaction with healthcare provider, including work recommendations.

## Conclusions

For physiotherapists, occupational therapists, and other professionals providing treatment for adults with upper limb MSDs, supporting patients to return to or remain in work is key component of holistic rehabilitation. This review identified three broad intervention strategies to support patients with work. We found that exercise interventions may have a positive effect on return to work, self-reported work functioning, and work productivity. Exercise interventions may be more effective that those incorporating multidisciplinary or ergonomic interventions; however, there was a lack of robust research for the latter interventions and we were unable to explore the impact key contextual factors, including of injury severity. Recommendations include development of a standardised classification for work-related musculoskeletal interventions and a core outcome set for musculoskeletal health and work.

We were unable to assess the impact of Fit Notes of other formal work guidance documentation because no studies were identified that evaluated these interventions for patients with upper limb conditions.

There was no apparent pattern between the intervention setting (healthcare or workplace) and diagnostic label (work-related or not) and the reported intervention effect. Contextual factors, including type of work, injury severity, sick leave policies, or benefits access, were not consistently reported and further exploration is warranted to determine the effect of different intervention strategies for different patient groups.

## Supplementary Information

Below is the link to the electronic supplementary material.

Supplementary file1 (DOCX 917 KB)

## Data Availability

The datasets generated during this review and other supporting material are available as supplementary files.
