# Supplementary material for: Effectiveness and Characteristics of Work Participation Interventions for Adults with Musculoskeletal Upper Limb Conditions: A Systematic Review
Source: J Occup Rehabil. 2024 Dec 5;35(4):741–66. doi: 10.1007/s10926-024-10251-6 (PMC12575510; doi:10.1007/s10926-024-10251-6)
Supplement: Supplementary file 1 — Supplementary file1 (DOCX 917 KB) [file 10926_2024_10251_MOESM1_ESM.docx]

# Supplementary material

# Appendix 1. PRISMA Checklist

| **Section and Topic** | **Item #** | **Checklist item** | **Location where item is reported** |
| --- | --- | --- | --- |
| **TITLE** | | |  |
| Title | 1 | Identify the report as a systematic review. | Title |
| **ABSTRACT** | | |  |
| Abstract | 2 | See the PRISMA 2020 for Abstracts checklist. | Abstract |
| **INTRODUCTION** | | |  |
| Rationale | 3 | Describe the rationale for the review in the context of existing knowledge. | Introduction |
| Objectives | 4 | Provide an explicit statement of the objective(s) or question(s) the review addresses. | Introduction |
| **METHODS** | | |  |
| Eligibility criteria | 5 | Specify the inclusion and exclusion criteria for the review and how studies were grouped for the syntheses. | Table 1 + Data synthesis and reporting |
| Information sources | 6 | Specify all databases, registers, websites, organisations, reference lists and other sources searched or consulted to identify studies. Specify the date when each source was last searched or consulted. | Search strategy + Figure 1 |
| Search strategy | 7 | Present the full search strategies for all databases, registers and websites, including any filters and limits used. | Search strategy + Appendix 2 |
| Selection process | 8 | Specify the methods used to decide whether a study met the inclusion criteria of the review, including how many reviewers screened each record and each report retrieved, whether they worked independently, and if applicable, details of automation tools used in the process. | Search strategy |
| Data collection process | 9 | Specify the methods used to collect data from reports, including how many reviewers collected data from each report, whether they worked independently, any processes for obtaining or confirming data from study investigators, and if applicable, details of automation tools used in the process. | Data extraction + Methodological quality assessment |
| Data items | 10a | List and define all outcomes for which data were sought. Specify whether all results that were compatible with each outcome domain in each study were sought (e.g. for all measures, time points, analyses), and if not, the methods used to decide which results to collect. | Table 1 + Tables 2-4 |
|  | 10b | List and define all other variables for which data were sought (e.g. participant and intervention characteristics, funding sources). Describe any assumptions made about any missing or unclear information. | Tables 2-4 |
| Study risk of bias assessment | 11 | Specify the methods used to assess risk of bias in the included studies, including details of the tool(s) used, how many reviewers assessed each study and whether they worked independently, and if applicable, details of automation tools used in the process. | Data extraction and methodological quality assessment |
| Effect measures | 12 | Specify for each outcome the effect measure(s) (e.g. risk ratio, mean difference) used in the synthesis or presentation of results. | Tables 5-7 |
| Synthesis methods | 13a | Describe the processes used to decide which studies were eligible for each synthesis (e.g. tabulating the study intervention characteristics and comparing against the planned groups for each synthesis (item #5)). | Data synthesis and reporting |
|  | 13b | Describe any methods required to prepare the data for presentation or synthesis, such as handling of missing summary statistics, or data conversions. | Data synthesis and reporting |
|  | 13c | Describe any methods used to tabulate or visually display results of individual studies and syntheses. | Data synthesis and reporting |
|  | 13d | Describe any methods used to synthesize results and provide a rationale for the choice(s). If meta-analysis was performed, describe the model(s), method(s) to identify the presence and extent of statistical heterogeneity, and software package(s) used. | Data synthesis and reporting |
|  | 13e | Describe any methods used to explore possible causes of heterogeneity among study results (e.g. subgroup analysis, meta-regression). | Data synthesis and reporting |
|  | 13f | Describe any sensitivity analyses conducted to assess robustness of the synthesized results. | Data synthesis and reporting |
| Reporting bias assessment | 14 | Describe any methods used to assess risk of bias due to missing results in a synthesis (arising from reporting biases). | Data extraction and methodological quality assessment |
| Certainty assessment | 15 | Describe any methods used to assess certainty (or confidence) in the body of evidence for an outcome. | Not applied |
| **RESULTS** | | |  |
| Study selection | 16a | Describe the results of the search and selection process, from the number of records identified in the search to the number of studies included in the review, ideally using a flow diagram. | Figure 1 + Appendix 3 |
|  | 16b | Cite studies that might appear to meet the inclusion criteria, but which were excluded, and explain why they were excluded. | Results + Appendix 3 |
| Study characteristics | 17 | Cite each included study and present its characteristics. | Study characteristics + Tables 2-4 + Appendix 5 |
| Risk of bias in studies | 18 | Present assessments of risk of bias for each included study. | Quality assessment and risk of bias + Figure 2 + Appendix 4 |
| Results of individual studies | 19 | For all outcomes, present, for each study: (a) summary statistics for each group (where appropriate) and (b) an effect estimate and its precision (e.g. confidence/credible interval), ideally using structured tables or plots. | Tables 5-7 |
| Results of syntheses | 20a | For each synthesis, briefly summarise the characteristics and risk of bias among contributing studies. | Work participation interventions + Figure 2 |
|  | 20b | Present results of all statistical syntheses conducted. If meta-analysis was done, present for each the summary estimate and its precision (e.g. confidence/credible interval) and measures of statistical heterogeneity. If comparing groups, describe the direction of the effect. | Work participation interventions + Figure 2 |
|  | 20c | Present results of all investigations of possible causes of heterogeneity among study results. | Work participation interventions + Figure 2 |
|  | 20d | Present results of all sensitivity analyses conducted to assess the robustness of the synthesized results. | Not applicable |
| Reporting biases | 21 | Present assessments of risk of bias due to missing results (arising from reporting biases) for each synthesis assessed. | Appendix 4 |
| Certainty of evidence | 22 | Present assessments of certainty (or confidence) in the body of evidence for each outcome assessed. | Tables 5-7 |
| **DISCUSSION** | | |  |
| Discussion | 23a | Provide a general interpretation of the results in the context of other evidence. | Discussion |
|  | 23b | Discuss any limitations of the evidence included in the review. | Strengths and limitations |
|  | 23c | Discuss any limitations of the review processes used. | Strengths and limitations |
|  | 23d | Discuss implications of the results for practice, policy, and future research. | Discussion + Conclusions |
| **OTHER INFORMATION** | | |  |
| Registration and protocol | 24a | Provide registration information for the review, including register name and registration number, or state that the review was not registered. | Abstract + Methods |
|  | 24b | Indicate where the review protocol can be accessed, or state that a protocol was not prepared. | Methods |
|  | 24c | Describe and explain any amendments to information provided at registration or in the protocol. | Registration reference |
| Support | 25 | Describe sources of financial or non-financial support for the review, and the role of the funders or sponsors in the review. | Abstract + Funding |
| Competing interests | 26 | Declare any competing interests of review authors. | Conflicts of interest |
| Availability of data, code and other materials | 27 | Report which of the following are publicly available and where they can be found: template data collection forms; data extracted from included studies; data used for all analyses; analytic code; any other materials used in the review. | Methods |

*From:*  Page MJ, McKenzie JE, Bossuyt PM, Boutron I, Hoffmann TC, Mulrow CD, et al. The PRISMA 2020 statement: an updated guideline for reporting systematic reviews. BMJ 2021;372:n71. doi: 10.1136/bmj.n71

# Appendix 2. Search strategy

Example search strategy designed in Medline. Terms were be filtered using title, abstract and keyword content. Subject headings were explored in each database and used as available. MeSH terms were searched as free text. Searches were limited to adult humans, where possible.

#### Population

Musculoskeletal ADJ disorder* OR musculoskeletal diseases (MeSH) OR musculoskeletal pain (MeSH) OR injur* OR wounds and injuries (MeSH)

AND

Upper ADJ limb OR upper ADJ extremit* OR upper extremity (MeSH) OR arm injuries (MeSH) hand (MeSH) OR hand* OR wrist* OR wrist injuries (MeSH) OR arm* OR arm (MesH) OR elbow* or elbow (MeSH) or elbow injuries (MeSH) OR shoulder* or shoulder (MeSH) or shoulder injuries (MeSH)

AND

#### Intervention

Program* OR rehab* OR rehabilitation (MeSH) OR training OR education* OR fit ADJ note* OR work ADJ report* OR self ADJ management OR advice OR vocational ADJ rehab*

AND

#### Outcomes

Work ADJ3 participation OR work (MeSH) return to work (MeSH) occupation OR employment (MeSH) OR unemployment (MeSH) OR sick ADJ leave OR work ADJ ability OR work ADJ performance

# Appendix 3. Data extraction items

| **Publication details** | **Research details** |
| --- | --- |
| Author  Year  Country  Region  Corresponding author details | Study design  Ethics details  Research paradigm  Study aims/research question  Study inclusion/exclusion criteria |
| **Clinical details** | **Intervention details** |
| Included conditions  Trauma/acquired  Surgical/non-surgical  Clinical setting and referral process  Clinicians involved | Setting (healthcare/workplace)  Content and format of the intervention  Intervention rationale  Dates of intervention |
| **Participant details** | **Occupational setting** |
| Number of participants (upper limb specific)  Age and range  Gender  Ethnicity  Other reported demographics | Work location  Sick pay entitlement/workers’ compensation |
| **Outcomes** | **Conclusions** |
| Outcome timepoints  Primary outcome  Other outcomes | Study conclusions  Limitations |

# Appendix 4. Excluded studies and reason for exclusion


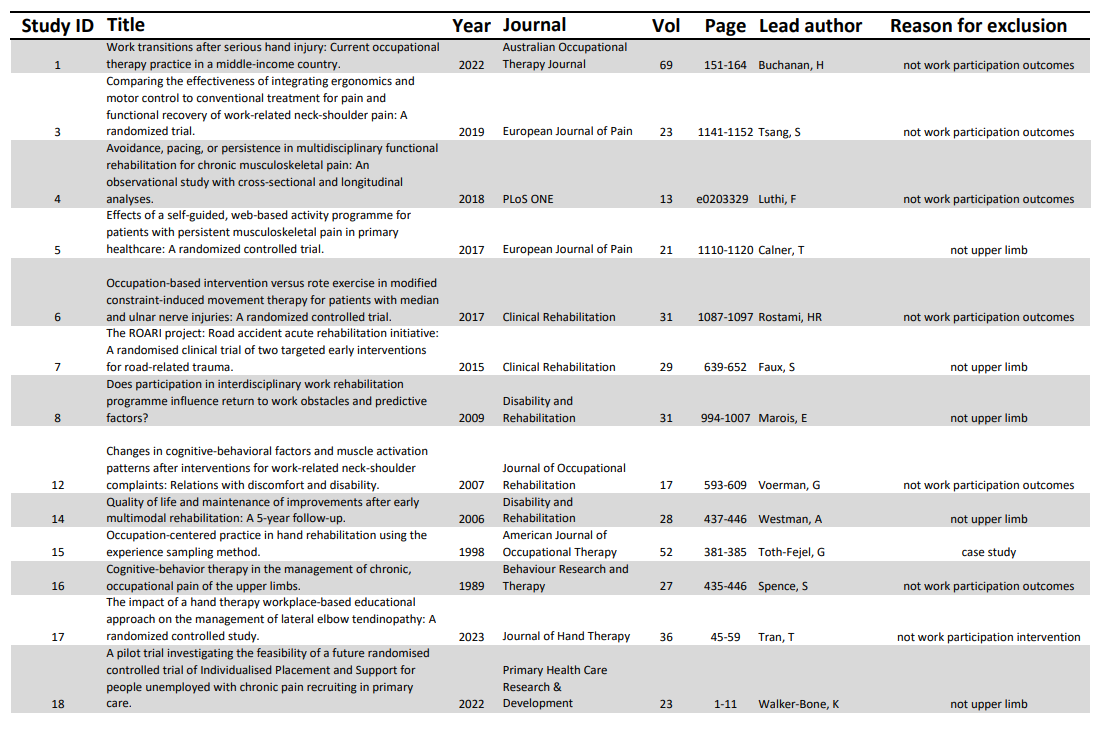


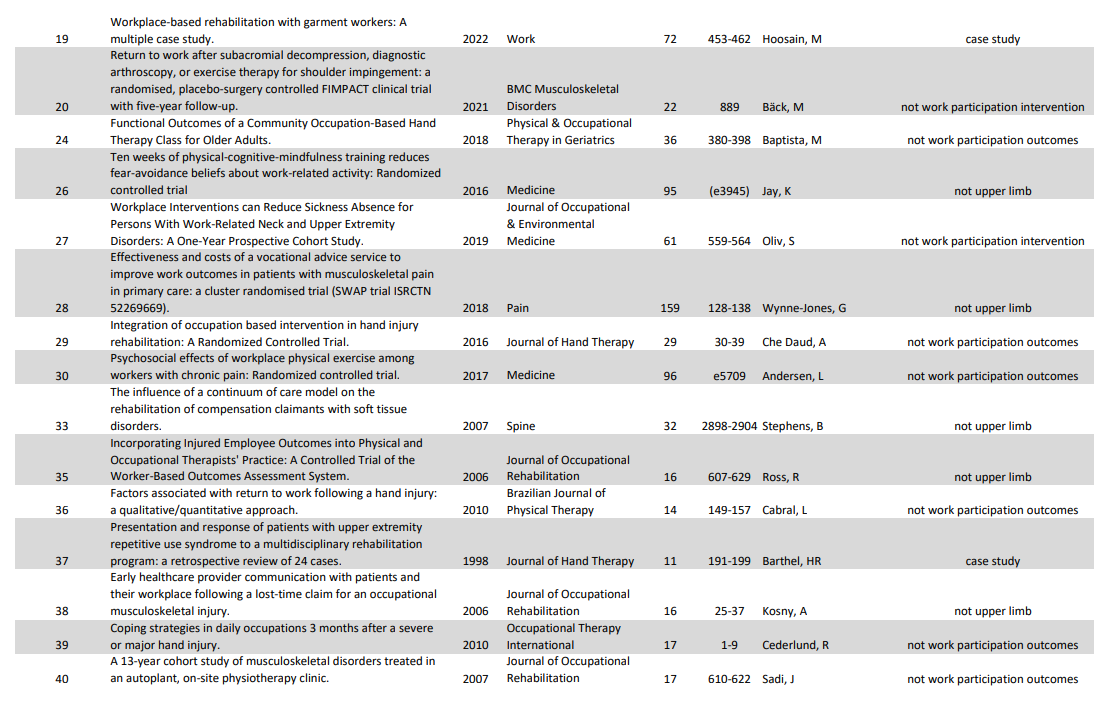


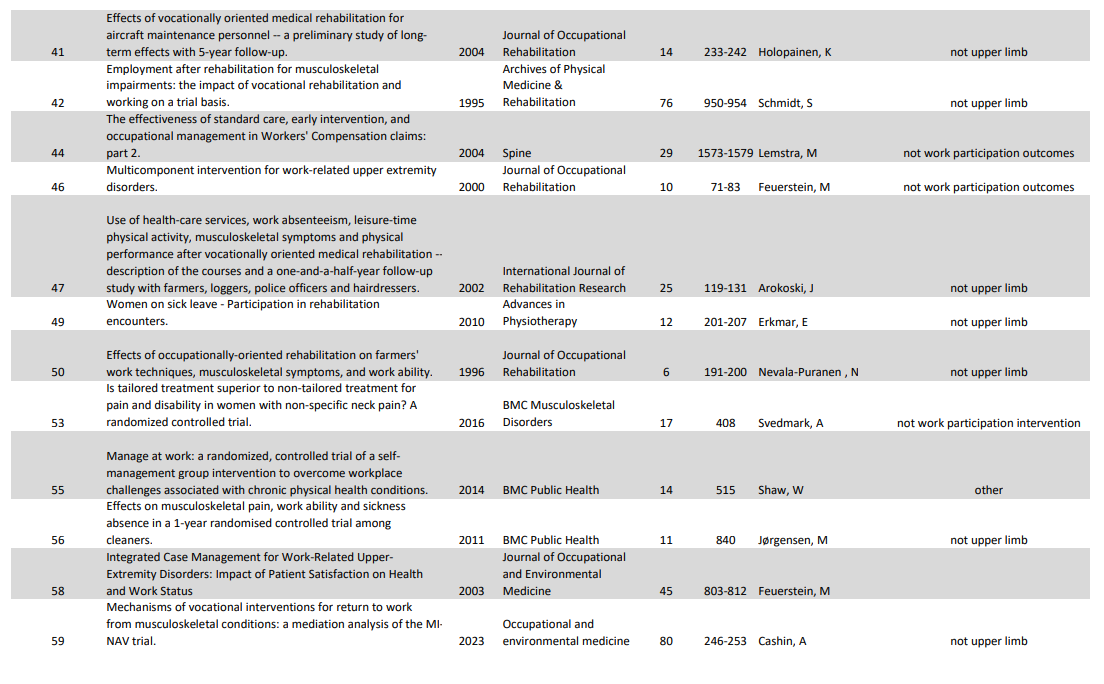


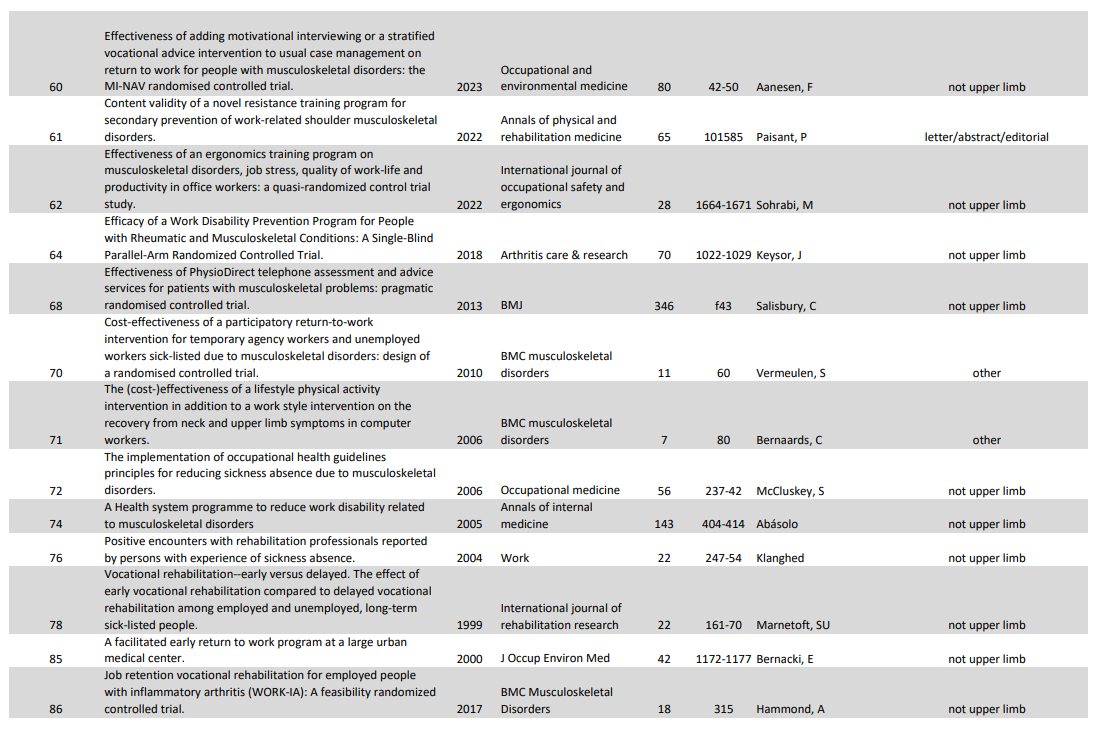


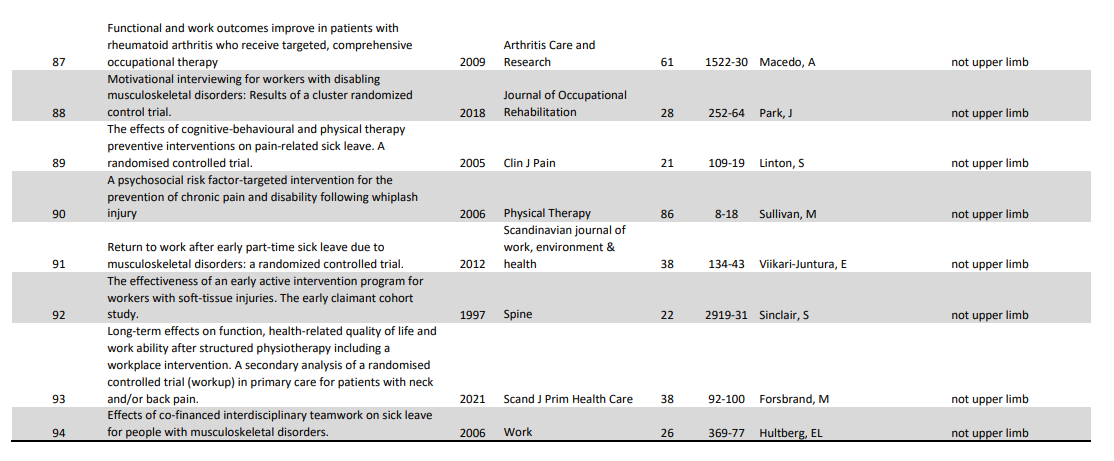


| Appendix 5. Quality assessment scoring | \|  \|  \| Clear cause / effect variables \| Baseline similarity \| Other treatment identical \| Control group \| Multiple measures of outcome \| Complete follow-up \| Identical outcome assessment \| Reliable outcome measures \| Appropriate statistical analysis \| \| --- \| --- \| --- \| --- \| --- \| --- \| --- \| --- \| --- \| --- \| --- \| \| Ekberg \| 1994 \|  \|  \|  \|  \|  \|  \|  \|  \|  \| \| Feuerstein \| 1993 \|  \|  \|  \|  \|  \|  \|  \|  \|  \| \| Harth \| 2008 \|  \|  \|  \|  \|  \|  \|  \|  \|  \| \| Storrø \| 2004 \|  \|  \|  \|  \|  \|  \|  \|  \|  \|   **B. Quasi-experimental studies**  (controlled, non-randomised) |
| --- | --- | --- | --- | --- | --- | --- | --- | --- | --- | --- | --- | --- | --- | --- | --- | --- | --- | --- | --- | --- | --- | --- | --- | --- | --- | --- | --- | --- | --- | --- | --- | --- | --- | --- | --- | --- | --- | --- | --- | --- | --- | --- | --- | --- | --- | --- | --- | --- | --- | --- | --- | --- | --- | --- | --- | --- |
| \|  \|  \| Randomisation \| Concealed allocation \| Baseline similarity \| Participant blinding \| Intervention-deliverer blinding \| Other treatment identical \| Outcome assessor blinding \| Identical outcome assessment \| Reliable outcome measures \| Complete follow-up \| Analysed and randomised \| Appropriate statistical analysis \| Appropriate trial design \| \| --- \| --- \| --- \| --- \| --- \| --- \| --- \| --- \| --- \| --- \| --- \| --- \| --- \| --- \| --- \| \| Blanquero \| 2020 \|  \|  \|  \|  \|  \|  \|  \|  \|  \|  \|  \|  \|  \| \| Cheng \| 2007 \|  \|  \|  \|  \|  \|  \| * \|  \|  \|  \|  \|  \|  \| \| Esmaeilzadeh \| 2014 \|  \|  \|  \|  \|  \|  \| * \|  \|  \|  \|  \|  \|  \| \| Hutting \| 2015 \|  \|  \|  \|  \|  \|  \| * \|  \|  \|  \|  \|  \|  \| \| Meijer \| 2006 \|  \|  \|  \|  \|  \|  \| * \|  \|  \|  \|  \|  \|  \| \| Monticone \| 2021 \|  \|  \|  \|  \|  \|  \| * \|  \|  \|  \|  \|  \|  \| \| Nemes \| 2013 \|  \|  \|  \|  \|  \|  \| * \|  \|  \|  \|  \|  \|  \| \| Shiri \| 2011 \|  \|  \|  \|  \|  \|  \|  \|  \|  \|  \|  \|  \|  \| \| So \| 2019 \|  \|  \|  \|  \|  \|  \| * \|  \|  \|  \|  \|  \|  \| \| Sundstrup \| 2014 \|  \|  \|  \|  \|  \|  \| * \|  \|  \|  \|  \|  \|  \| \| Thorndal Moll \| 2018 \|  \|  \|  \|  \|  \|  \|  \|  \|  \|  \|  \|  \|  \| \| van den Heuvel \| 2003 \|  \|  \|  \|  \|  \|  \|  \|  \|  \|  \|  \|  \|  \|   **A. Randomised controlled trials** |  |
|  | \|  \|  \| Clear inclusion criteria \| Condition measured reliably and standardised \| Valid method for identification of condition \| Consecutive participant inclusion \| Complete inclusion of participants \| Key demographics reported \| Key clinical information reported \| Outcomes or follow-up results reported \| Clinic/location clearly described \| Appropriate statistical analysis \| \| --- \| --- \| --- \| --- \| --- \| --- \| --- \| --- \| --- \| --- \| --- \| --- \| \| Bean \| 2017 \|  \|  \|  \|  \|  \|  \|  \|  \|  \|  \| \| Schakenraad \| 2004 \|  \|  \|  \|  \|  \|  \|  \|  \|  \|  \| \| Shaw \| 2008 \|  \|  \|  \|  \|  \|  \|  \|  \|  \|  \| \| Sherrod \| 2014 \|  \|  \|  \|  \|  \|  \|  \|  \|  \|  \| \| Voss \| 2019 \|  \|  \|  \|  \|  \|  \|  \|  \|  \|  \|   **D. Case series** |
| \|  \|  \| Qualitative approach appropriate \| Qualitative data collection methods appropriate \| Qualitative sampling methods & sample appropriate \| Qualitative findings adequately derived \| Qualitative interpretations substantiated by data \| Coherence between qualitative data sources \| Quantitative sample strategy appropriate \| Sample representative of target population \| Measurements appropriate \| Low risk of non-response bias \| Appropriate statistical analysis \| Adequate rationale for mixed methods design \| Different study components effectively integrated \| Outputs adequately interpreted \| Divergences and inconsistencies addressed \| \| --- \| --- \| --- \| --- \| --- \| --- \| --- \| --- \| --- \| --- \| --- \| --- \| --- \| --- \| --- \| --- \| --- \| \| Hutting \| 2017 \|  \|  \|  \|  \|  \|  \|  \|  \|  \|  \|  \|  \|  \|  \|  \|   **C. Mixed methods studies** |  |
|  | \|  \|  \| Well defined question \| Description of alternatives \| Alternative costs and outcomes identified \| Established clinical effectiveness \| Costs and outcomes measured accurately \| Costs and outcomes valued credibly \| Costs and outcomes adjusted for differential timing \| Incremental analysis of costs and consequences \| Sensitivity analysis included \| Results include all issues of interest to users \| Results generalisable to the review setting \| \| --- \| --- \| --- \| --- \| --- \| --- \| --- \| --- \| --- \| --- \| --- \| --- \| --- \| \| Bernaards \| 2011 \|  \|  \|  \|  \|  \|  \|  \|  \|  \|  \|  \|   **E. Economic evaluations** |
| **Key** |  |
| \| Yes \| No \| Unclear \| Not applicable \| \| --- \| --- \| --- \| --- \| \|  \|  \|  \|  \|   All studies were assessed in relation to work-related outcomes. If more than one outcome was relevant to this review, the figure shows the highest grading.  * Solely patient reported outcomes.  A, B, D, E Johanna Briggs Institute Critical Appraisal Tools.  C Mixed methods assessment tool. |  |

# Appendix 6. Description of the interventions using TIDieR (template for intervention description and replication)

## Multimodal or multidisciplinary interventions

| **Author, year** | **Intervention name** | **Rationale** | **Control treatment** | **Intervention(s) materials and procedures** | **Timescales** | **Duration** | **Professions involved** | **Fidelity and adherence** | **Location** |
| --- | --- | --- | --- | --- | --- | --- | --- | --- | --- |
| Feuerstein  1993 | Multi- component work rehabilitation programme | Rochester Model of Work disability: work disability is a complex interaction of medical status, physical capabilities, work demands and psychological factors. All components need to be addressed for rehabilitation | *Usual care;*  managed by physician with access to physiotherapy, hand therapy, exercise, chiropractic treatment, rehabilitation counselling and pain treatment | - Warm-up (30 mins) - Physical conditioning (55 mins) - Work simulation (55 mins) - Job related pain and stress management (45 mins) - Ergonomic consultations - Vocational counselling - Individualised home exercise programm3 | Daily programme | 4-6 weeks | Exercise physiologist or physiotherapist, psychologist, work conditioning technician, vocational counsellor | Fidelity: NR  Adherence: NR | Healthcare (occupation-al health) |
| Ekberg  1994 | Early multi-  disciplinary rehabilitation | Structured, multidisciplinary rehabilitation would improve outcomes | *Usual care*; physiotherapy, medication, rest and sick leave | - Individualised physical training - Information provision and education on health promotion, ergonomics, smoking cessation and nutrition - Social interaction - Workplace visits | 2 hours per day, 4 days per week. 5-8 per group | 8 weeks | Physiotherapist | Fidelity: NR  Adherence: 2 (4%) did not complete intervention | Healthcare (industrial health care unit) |
| Schakenraad  2004 | Multi-  disciplinary rehabilitation programme | Non-specific upper limb conditions require multi-disciplinary and multi-factorial approaches | N/A | Multidisciplinary treatment:   - Graded activity - Stress coping strategies, including cognitive techniques, health education, medication reduction, relaxation, goal setting and return to work planning - Phased RTW | 13 full days, each with 4 treatment sessions of 1.5 hours, in groups of <9. 2.5 days of phased RTW | 3 weeks | Psychologist, physiotherapist, orthopaedic surgeon, occupational therapist | Fidelity: all compliant with treatment protocol  Adherence: NR | Healthcare (occupation-al health) |
| Storrø  2004 | Multi- disciplinary intervention | Multi-disciplinary interventions may improve health through different dimensions; physical abilities body awareness, coping skills and pain understanding | *Usual care;* including referral to physiotherapist, chiropractor | Group sessions and exercises programmes:   - Exploration of pain perception, self-confidence, reduction of fear-avoidance behaviour, and skills to cope with pain - Individualised physical exercises for posture, aerobic capacity, strength, flexibility - Aerobic and strength training, including swimming   Relaxation and body awareness training | 3 group sessions per week (2 hours each)  8-10 per group | 4 weeks | Medical doctor, physiotherapist, psychologist | NR | Healthcare facility within a fitness centre |
| Meijer  2006 | Multi-  disciplinary return to work programme | Sick-leave has negative psychological impacts on employees; multi-disciplinary programme will improve work outcomes | *Usual care*; including workplace or healthcare-based treatment | Outpatient training programme:   - Psychological sessions - Relaxation - Physical sessions – strength, endurance, aerobic fitness using graded activity - Pain education - Sporting activities, e.g. bowling - Return to work sessions and feedback | 13 days (09:00-17:00), with 4 treatment sessions of 1.5 hours, in groups of 8  5 RTW sessions | 2 months | Physiotherapist, psychologist, medic, occupational therapist | Fidelity: standardised protocol, 75% compliance  Adherence: 7% UC, 9% intervention did not complete | Healthcare |
| Harth  2008 | Patient-  orientated hand rehabilitation programme | Rehabilitation focused on individual patient needs and concerns will improve outcomes. | *Usual care*, not further defined | - Two individualised physiotherapy and occupational therapy sessions with goal setting based on individual priorities and including work-related activities, pain management and addressing psychological issues - Group sports activities - Gym activities - Interaction between rehabilitation team and employer | Daily programme | 5-6 weeks | Doctor, occupational therapist, physiotherapist, sports therapist, psychologist, social worker, vocational rehabilitation manager | Fidelity: NR.  Adherence: NR | Healthcare (inpatient) |
| Shaw  2008 | Workplace-  based return to work program | Workplace-based programmes enable workers to remain productive in the workplace while recovering from injury, and engage all stakeholders in the return to work process. | N/A | Worker and supervisor manage early shoulder symptoms (e.g. ergonomic and schedule changes). If persist, worker presents to occupational health, who refers OT for:   - Interview, functional assessment, workplace assessment - Individualised RTW plan e.g. amended duties, amended schedule, job rotation, - Reassessment and reintegration to more demanding duties/ hours, with support from co-workers - Regular reassessments and plan modification (+/- medical evaluations). If deemed not able to return to pre-injury job - placed elsewhere - RTW plan closed once resumed regular duties and hours (preinjury job or placement job) | Individualised | NR | Supervisor, employer, occupational health nurse, physiotherapist, occupational therapist, ergonomist, occupational health physician, safety specialist | Fidelity: Mandatory process within the employing organisation.  Adherence:  7% unable to return to work (participate in the workplace-  based programme) | Workplace: clinic located in the workplace |
| Bernaards  2011 | Work style and physical activity training | Precaution Adoption Process Model of behaviour change: reflecting and planning for future action.  Dynamic model of workload: increasing capacity and decreasing workload restores imbalance in health. | *Usual care;* including physiotherapy or medical interventions.  All 3 groups had ‘breaks and exercise reminder software’. | *Workstyle*   - Information on neck and upper limb symptoms and risk factors - Importance of breaks and movement - Workplace adjustments - Recognising work stress   *Workstyle + physical activity*  As above, plus:   - information on importance of physical activity in symptom reduction - Physical activity planning, exploring potential barriers and solutions - Resistance band upper limb exercise   programme | 6 interactive group sessions (max 4-10 per group)  60 minutes for each workstyle session, additional 30 minutes for physical activity | 6 months | Trained counsellor | Fidelity: Protocol for each session.  Adherence:  7% Workstyle,  13% Workstyle + physical activity,  6% usual care did not complete | Workplace during work time |
| Nemes  2013 | Rehabilitation therapy |  | *Usual care*, not further described  Both groups received medical treatment, including anti-inflammatories, muscle relaxants | Home-adapted rehabilitation programme  - Balanced professional activities, including breaks at work | 10 rehabilitation sessions every 6 months | 2 years | Rehabilitation professionals not further described | NR | Healthcare |
| Hutting  2015, 2017 | Self-  management intervention with E-Health module | Self-management is used in chronic disease care to improve self-efficacy, wellness behaviours, and characteristics of the work environment | *Usual care;* no restrictions for other treatments | Group sessions, including SMART goal-setting:   - 1: Dealing with a chronic disability, living and working with upper extremity complaints, workload and work capacity, what is self-management, introduction to the eHealth module - 2: Discussion of eHealth module, core qualities, time management - 3: Dealing with pain and fatigue, stress management, muscle relaxation - 4: Healthy lifestyle, nutrition, exercise and sports, including use of facilities - 5: Communication skills, working with others, asking for help - 6: Dealing with negative emotions, positive thinking, making a mind map   Self-directed e-learning: e-health, self-management, upper extremity complaints, possible solutions, further reading | Weekly group sessions (2.5 hours each). 4-12 per group | 6 weeks  eHealth module available for up to 12 months | Groups coordinated by moderator -unclear whether clinician or other professional | Fidelity: NR  Adherence:  9 (14%) did not attend the group sessions. Of those who attended, 92% completed ≥4 sessions | Not clear where the group sessions were held. Assume healthcare. No response from authors |
| Bean  2017 | Multi-  disciplinary rehabilitation programme | Multidisciplinary rehabilitation programmes for musculoskeletal conditions show improved physical function, pain, ability to return to work, and reduce emotional distress. Early participation at work, in any capacity, helps maintain physical conditioning, quality of life, and function | N/A | - SMART goal setting - Education on diagnosis, treatment, prognosis - Team members met regularly to discuss, with the patient encouraged to participate in clinical decision-making. - PT: individualised treatment including modalities to improve pain, general conditioning for strength and endurance, manual therapy, and stretching. - OT: functional activities related to work, home and leisure to increase strength, endurance and function. Explored barriers to progress, provided information on the difference between hurt and harm, good body postures, and lifting techniques.   Encouraged to return to modified or regular duties as appropriate. | 3 sessions per week, 45 mins with PT and 45 mins with OT | 22% 6-8 weeks  8% 8-10 weeks  60% >12 weeks | Orthopaedic surgeon, physiotherapist, occupational therapist | NR | Healthcare |
| Thorndal Moll  2018 | Multi-  disciplinary intervention | Multidisciplinary rehabilitation optimises return to work and reduces pain and disability | *Usual care;*  rheumatology and PT assessment and imaging. Education, reassurance and home exercise programme. Follow-up with rheumatologist and physiotherapist to discuss imaging and exercise adherence. Advised to return to work when possible. Advised to consult GP for any addition input | As usual care, plus:   - Individual case manager - initial standardised interview on work history, private life, pain and disability. Return to work rehabilitation plan - Consultation with a psychologist, if needed - Case manager discussed relevant matters at regular team conferences +/- employer. Not attended by the patient. - If return to original work was considered impossible, an alternative plan to remain in work was made, or a meeting was arranged with the municipality’s social service centre. | Met case manager at 3-4 weeks. Further meetings, depending on need | Medical and PT sessions at 2 weeks, 3-6 weeks and 12 weeks (both groups) | Physiotherapist, Rheumatologist, Case manager (social worker or occupational therapist), Psychologist | Fidelity: Team supervision every 1-2 months to ensure standardisation.  Adherence: NR | Healthcare, within a group session in the workplace, if needed |
| Voss  2019 | Inter-  disciplinary, work rehabilitation programme | Interdisciplinary programs target psychological, social and physical aspects of an injury for improved work outcomes | N/A | Interdisciplinary approach with an emphasis on returning the worker to their prior occupation. Including:   - Work simulation - Cardiovascular activity - Overall body strengthening - - Psychological services, job coaching, ergonomic evaluation and transitional work services (as appropriate). | 3-5 days per week (1-3 hrs per session). | 3-72 visits | Physiotherapist, occupational therapist, case manager, licensed athletic trainer, psychologist, vocational counsellor. | Fidelity: NR  Adherence: NR | Healthcare: non-for-profit healthcare system |

## Ergonomic interventions

| **Author, year** | **Intervention name** | **Rationale** | **Control treatment** | **Intervention(s) materials and procedures** | **Timescales** | **Duration** | **Professions involved** | **Fidelity and adherence** | **Location** |
| --- | --- | --- | --- | --- | --- | --- | --- | --- | --- |
| Shiri  2011 | Ergonomic workplace-  based intervention | Workplace-based tailored intervention will be more effective in reducing productivity loss at work than traditional disease and disability management | *Best practice usual care*, not further described | Workplace-based ergonomic adaptations:   - Physician contacted supervisor to discuss work accommodations - Workplace visit from occupational PT to find ergonomic improvements, including work posture, force requirements, pace and breaks   Findings discussed with the employee and supervisor, who had the final decision and financed any changes | Single visit from PT, Other timescales NR | NR | Physiotherapist, physician, employer | NR | Workplace: clinics located in the place of work |
| Sherrod  2014 | Ergonomic intervention and chiropractic care | Psychosocial components of care and adoption of measures to reduce risk exposures are important in successful preventative management of work-related musculoskeletal disorders | N/A | Combined adjunctive chiropractic care (usual care), plus:   - Ergonomic intervention of: assessment, team training, computer workstation retrofits and designs, installation of furniture and equipment, and space planning logistics. | Weekly chiropractic care, plus 2 hours of ergonomic training | 16 weeks (or longer if needed) | Chiropractor, ergonomist | NR | Workplace |
| Esmaeilzadeh  2014 | Ergonomic training | Improved condition management through training on workplace ergonomics, workstation self-assessment and adjustment | Treatment not reported | - Group ergonomic training programme - Booklet on office ergonomics, including risk factors for upper limb conditions, importance of prevention, workstation adjustments and workplace exercises   Individualised workstation evaluation, with monthly re-evaluations | Two 90-minute group sessions (max 20 people) | NR | Trained ergonomist | Fidelity: NR  Adherence: 5 (7%) intervention, 7 (10%) control did not complete. | Workplace |
| So 2019 | Ergomotor training | Ergomotor intervention training may influence both physical and psychosocial aspects of work-style and work related musculoskeletal disorders | *Usual care;* physiotherapy, including electrophysical modalities | Individualised programme including:   - Workplace-based ergonomics knowledge transfer - Biofeedback motor control facilitation - Tailor-made neck and shoulder motor re-education exercises   Implementing learning into daily work practice | 16 sessions (60 minutes each) | 12 weeks | Trained therapist | Fidelity: NR  Adherence: 14% intervention, 16% control did not complete | Healthcare |

## Exercise interventions

| **Author, year** | **Intervention name** | **Rationale** | **Control treatment** | **Intervention(s) materials and procedures** | **Timescales** | **Duration** | **Professions involved** | **Fidelity and adherence** | **Location** |
| --- | --- | --- | --- | --- | --- | --- | --- | --- | --- |
| Van den Heuvel 2003 | Software programme stimulating breaks and exercise | Changes to the temporal pattern of movement will reduce prevalence of neck and upper limb disorders | Booklet with general information on neck and upper limb disorders, plus workplace ergonomic assessment and adjustment. Provided to all 3 groups | *Breaks*   - Software initiated work breaks of 5 mins for every 35 mins continuous usage, and microbreak after every 5 mins continuous usage   *Breaks + Exercise*  As above, with exercises presented on the screen during each work break | During working day | 8 weeks | Automated electronic intervention | NR | Workplace |
| Cheng  2007 | Workplace-  based work hardening program | Workplace-based interventions enable injured workers to test work ability in real-world situations. This optimised management strategies and stakeholder involvement | *Usual care;* clinic-based work hardening training, comprising upper limb mobilisation activities, strength and endurance training, and work simulation. | Workplace-based programme   - Job coach assigned to each worker, who contacted the worker’s supervisor to arrange suitable work tasks as treatment - Biomechanics and ergonomic education, plus booklet - Standardised shoulder exercises: stretches, control, strengthening - Individualised job specific training activities | 3 sessions per week | 4 weeks | Job coach, other rehabilitation professionals not reported | Fidelity: NR  Adherence:  4% usual care, 5% intervention did not complete | Intervention: workplace and control: healthcare |
| Sundstrup  2014 | Strength training | Work ability is the relationship between worker capacity and work demands: strength training will have positive effects on work ability | Ergonomic training and education, targetted to slaughterhouse workers | Strength training:   - Supervised high intensity strength training for shoulder, arm and hand Progressively increased from 20 repetition maximum (RM) to 8 RM using principles of periodisation and progressive overload   Received portable exercise equipment for home training in case of absence from work (e.g. vacation). | 3 x 10 minute sessions per week | 10 weeks | Trained instructor | Fidelity: NR  Adherence: Strength training 80%, mean 2.4/3 sessions per week | Workplace |
| Blanquero  2020 | ReHand tablet application | Telerehabilitation supports recovery of physical function for patients with musculoskeletal conditions. Telerehabilitation uses games as a rehabilitation tool to improve pain and functional outcomes | *Usual care;* home exercise programme on paper. Prescribed 2x per day for 20-30 mins  Both groups received OT and PT interventions, including splinting, manual therapy, electrotherapy, active exercises and sensorimotor training. | Tablet-based application (ReHand app) for home exercise programme:  - All exercises performed using the touchscreen and adjusted to individual pain-free range  - Involves wrist, finger, thumb movements | Home exercises on tablet 2x per day for 20-30 mins.  OT and PT for both groups): 3x per week, for 30- 60 mins | 4 weeks | Physiotherapist, occupational therapist | Fidelity: NR  Adherence:  18% did not complete intervention | Healthcare delivered service, intervention used at home |
| Monticone  2021 | Functional/  task-oriented exercises | Task-oriented exercises generate positive attitudes towards active training and recovery of physical performance. They improve functional outcomes and allow faster return to activities | *Usual care;* mobilisation within 1 week of surgery, strengthening, stretching, postural control, and sling care information. Encouraged to perform home exercises | As usual care group, plus:  - PT: Task-oriented exercises based on individualised job activities. Plus exercises for dexterity, balance, and other functional demands  - OT: Sling care information, ergonomic activities based on individualised pre-injury work activities | PT: 3x individual 60min sessions per week.  OT: 1x per week for 60min.  (both groups) | 12 weeks | Physical medicine and rehabilitation doctor, occupational therapist, physiotherapist | Fidelity: Standard treatment manual and fidelity check after each session. No crossover.  Adherence: 90-95% for both groups | Healthcare |

NA – not applicable. NR – not reported. PT – physiotherapy. OT – occupational therapy

*From:* Hoffmann T, Glasziou P, Boutron I, Milne R, Perera R, Moher D, Altman D, Barbour V, Macdonald H, Johnston M, Lamb S, Dixon-Woods M, McCulloch P, Wyatt J, Chan A, Michie S. Better reporting of interventions: template for intervention description and replication (TIDieR) checklist and guide. BMJ. 2014;348:g1687.
